# Distribution of Toxic and Essential Elements in Autopsy Organs of Subjects Living in South-Eastern Poland

**DOI:** 10.3390/ijms27062585

**Published:** 2026-03-11

**Authors:** Wojciech Flieger, Przemysław Niedzielski, Michał Flieger, Zofia Wojciechowska, Aleksandra Proch, Jędrzej Proch, Alicja Forma, Andrzej Torbicz, Dariusz Majerek, Grzegorz Teresiński, Jacek Baj, Eliasz Dzierżyński, Jolanta Flieger

**Affiliations:** 1 Department of Plastic Surgery, Saint John of Dukla Oncology Centre of the Lublin Region, Jaczewskiego 7, 20-090 Lublin, Poland; wflieger@cozl.pl (W.F.); idziarzhynski@cozl.pl (E.D.); 2 Institute of Health Sciences, John Paul II Catholic University of Lublin, Konstantynów 1 H, 20-708 Lublin, Poland; 3 Department of Analytical Chemistry, Faculty of Chemistry, Adam Mickiewicz University, Uniwersytetu Poznańskiego 8, 61-614 Poznań, Polandzofia.wojciechowska@amu.edu.pl (Z.W.); aleksandra.proch@amu.edu.pl (A.P.); jedrzej.proch@amu.edu.pl (J.P.); 4 Doctoral School, Medical University of Lublin, Aleje Racławickie 1, 20-059 Lublin, Poland; 58293@umlub.edu.pl; 5 Department of Correct, Clinical and Imaging Anatomy, Medical University of Lublin, Ul. Jaczewskiego 4, 20-090 Lublin, Poland; alicja.forma@umlub.edu.pl; 6 Department of Analytical Chemistry, Medical University of Lublin, Chodźki 4a (Collegium Pharmaceuticum), 20-093 Lublin, Poland; andrzej.torbicz@umlub.edu.pl; 7 Department of Applied Mathematics, University of Technology, 20-618 Lublin, Poland; d.majerek@pollub.pl; 8 Department of Forensic Medicine, Medical University of Lublin, Jaczewskiego 8b, 20-090 Lublin, Poland; grzegorz.teresinski@umlub.edu.pl

**Keywords:** toxic metals, essential elements, trace elements, bioaccumulation, multi-elemental analysis, human tissues, environmental exposure

## Abstract

Chronic exposure to heavy metals poses significant health risks. This study analyzed the concentrations of toxic (Cr, Pb, Cd, Ni) and essential (Cu, Zn, Se, Mn) elements in autopsy samples (the frontal pole area of the brain, the 6th intercostal space of the liver, and lungs (average of left and right lung samples) from 45 residents of South-Eastern Poland using ICP-MS. The aim was to determine the average body burden and organ-specific accumulation in a moderately industrialized region. HDBSCAN clustering revealed highly homogeneous elemental profiles, suggesting a unifying influence of local environmental factors. The liver acted as a metabolic hub, showing preferential sequestration (*p* < 0.0001) of essential elements (Zn, Se, Mn, Cu) regulated by homeostatic mechanisms. Toxic metals exhibited ‘metabolic trap’ patterns, particularly Cd and Pb in the liver and Cr in the lungs, due to their long biological half-lives. Strong positive correlations (Se–Zn, Se–Cu) indicated integrated antioxidant responses, while toxic pairs (Cr–Ni, Pb–Cd) suggested shared exposure pathways and molecular mimicry via transporters such as DMT1. Results confirmed long-term bioaccumulation, with toxic elements in the brain remaining below 0.25 µg/g. In the lungs, the accumulation hierarchy (Pb > Mn > Cd > Cr) reflected inhalation exposure. These findings emphasize the role of organ-specific sequestration in assessing long-term environmental exposure.

## 1. Introduction

Essential trace elements include zinc (Zn), copper (Cu), iron (Fe), manganese (Mn), selenium (Se), and chromium (Cr), which are crucial for enzymatic and metabolic functions at low concentrations but can be toxic when exposed to high doses [1,2]. These elements act as structural components or cofactors of enzymes, responsible for functions such as cellular respiration, gene expression, and the fight against oxidative stress [3,4,5]. It is known that approximately half of enzymes contain a metal cofactor to fulfill their functions [6,7,8].

In contrast, toxic metals (biologically insignificant), such as cadmium (Cd), lead (Pb), mercury (Hg), arsenic (As), and aluminum (Al), have no physiological functions and can be highly toxic even at low dose concentrations [9,10,11]. For example, lead (Pb) and mercury (Hg) are considered toxic at blood concentrations exceeding 10 µg/dL, and cadmium (Cd) above 5 µg/L [12,13]. It should be noted that elements such as cadmium (Cd), lead (Pb), mercury (Hg), arsenic (As), and aluminum (Al) are toxic systemically, meaning they affect organs distant from the site of entry [8].

Human tissues exhibit specific affinities for heavy metals. According to the examination of 150 Korean subjects (aged 12–87), the metabolic organs exhibit an affinity for metals such as cadmium (Cd), mercury (Hg), manganese (Mn), molybdenum (Mo), tin (Sn), and zinc (Zn), while aluminum (Al), chromium (Cr), and silicon (Si) show a preference for tissues exposed to the exterior [14]. Mineralized tissues, including teeth and bones, are the primary site of lead (Pb) deposition, where approximately 90% of the total body burden of this metal accumulates, increasing with age [15].

The metal content in tissues vary depending on the population studied. A study of a population from the province of Tarragona (Spain) showed that the median accumulation of cadmium (Cd) in the renal cortex was 10.8 µg/g wet weight [16]. Other sources from different countries report higher values of 15.28 µg/g (Turkey) [17] and 12.9 µg/g (Sweden) [18].

A 2018 study by Piotr Rzymski et al. [19] demonstrated that material from miscarriages in women (*n* = 20) from urban populations showed cadmium (Cd) levels that were over 1.5 times higher and lead (Pb) levels that were over twice as high as those collected from women living in rural areas (*p* < 0.05). The authors of the study indicate that human exposure to toxic factors in urban environments is elevated. The main route of exposure to toxic metals is inhalation of air polluted with suspended particulate matter (PM) with elements adsorbed on the surface. Also, a study by Servet Birgin Iritas et al. [10] conducted in Turkey (Ankara) showed that lead (Pb) levels in autopsy liver tissues in urban residents (*n* = 119) were significantly higher than in people living in suburbs or rural areas. The study by Gunawarden et al. [20] also confirms this relationship. A comparative analysis showed significantly higher cadmium (Cd, zinc (Zn), selenium (Se) levels in urban residents (*n* = 13) compared to rural residents (*n* = 18).

The levels of elements in various tissues and body fluids are not constant. They depend on factors such as place of residence, which influences environmental exposures, as well as dietary habits, smoking, and alcohol consumption. It has been observed that in individuals with alcoholism, a decrease in Co, Cu, Mg, and Mn was observed, accompanied by an increase in Fe in the liver [21]. The lungs are characterized by increased accumulation of chromium (Cr), aluminum (Al), and silicon (Si), reflecting inhalation exposure from atmospheric air [14]. Tobacco smok ing causes significant and multidirectional changes in the elemental composition of the respiratory tissues (lungs and bronchi), manifested mainly by the bioaccumulation of toxic metals: aluminum (Al) (almost three times higher than in non-smokers (18 µg/g vs. 6.7 µg/g wet weight), chromium (Cr): in the lung tissue of smokers it is about 6.4 µg/g, while in non-smokers it is only 2.2 µg/g, arsenic (As): in the lungs of smokers it is about twice as high as in the control group (0.43 ng/g vs. 0.28 ng/g wet weight), barium (Ba): an extremely high, as much as 7.5-fold increase in the barium concentration in the lungs of smokers was recorded (0.015 µg/g vs. 0.002 µg/g) [22]. Tobacco smoking is considered the main source of Cd in the lungs, which is of critical importance in the pathogenesis of lung diseases due to extremely long half-life (16–30+ years) [23,24].

Literature sources indicate that hip and knee joint replacements introduce various metal materials into the body [25]. The surfaces of these implants can release degradation products due to factors such as friction, corrosion, and the presence of free metal ions. Depending on the composition of the prosthesis, patients may be exposed to certain metal alloys, including chromium (Cr), cobalt (Co), nickel (Ni), and titanium (Ti), as well as aluminum (Al), vanadium (V), and molybdenum (Mo). High concentrations of these metals have been found in tissues surrounding the implant, such as the joint capsule. Furthermore, implant degradation can lead to systemic exposure, leading to increased metal concentrations in body fluids and distant organs, including the brain, liver, spleen, and abdominal lymph nodes. Therefore, the examination of tissues collected during autopsy from people with implants is, on the one hand, valuable for assessing the safety of implant materials, but on the other hand it should be taken into account as an exclusion criterion in metallomic studies.

The examination of autopsy tissues is crucial for advancing the field of toxicology. However, it is necessary to create databases that detail regional differences in metal accumulation within internal organs. Most research tends to concentrate on individual metals, but in reality, the body is exposed to mixtures of elements that can have synergistic or antagonistic effects. Analysis of metals in postmortem material faces significant challenges, including the lack of reference values. There is an urgent need to develop standardized postmortem reference ranges that would distinguish physiological background from toxicologically significant concentrations (e.g., in cases of suspected poisoning).

The selection of specific autopsy organs for this study was dictated by their distinct physiological roles and varying mechanisms of metal accumulation. The liver is the primary storage site for essential trace elements like copper (Cu), zinc (Zn), and manganese (Mn); it is also the organ responsible for biotransforming and detoxifying toxic metals. In contrast, the brain has high energy demands and a high lipid content, which makes it particularly vulnerable to oxidative stress caused by toxic metals. Furthermore, the lungs and bronchial tissues are directly exposed to airborne toxins and suspended particulate matter containing adsorbed metals. As a result, metal accumulation in the lungs directly reflects the deposition of atmospheric particles. Analyzing this specific set of organs provides a comprehensive overview of both systemic distribution and localized environmental impact, making it crucial to establish the baseline concentrations of both toxic and essential elements within the studied population.

Therefore, the aim of this study was to examine the accumulation of five selected metals: chromium (Cr), lead (Pb), cadmium (Cd), manganese (Mn), and nickel (Ni). These metals are persistent environmental pollutants that can bioaccumulate, leading to tissue and organ damage. Toxic metal levels, along with selected essential elements (Se, Zn, Cu), were examined in autopsy tissues collected from the liver, brain (including the frontal lobe), and air passages (lungs and bronchi).

## 2. Results

### 2.1. Elemental Concentration in the Frontal Pole Area of the Brain

Brain tissue from the frontal pole was analyzed for metals (Mn, Cu, Zn, Cr, Pb, Cd, Ni) and metalloid (Se) using ICP-MS. Descriptive statistics based on the measured data are summarized in Table 1. Across all analyzed elements, the distributions of concentrations in brain tissue deviate significantly from normality, as indicated by the Shapiro–Wilk test (*p* < 0.05) with Holm correction (all adjusted *p*-values < 0.001). As shown in Table 1, these distributions are characterized by substantial positive skewness and high kurtosis for most variables, reflecting strongly right-skewed patterns with heavy tails and the presence of extreme values. These results indicate pronounced distributional asymmetry and heterogeneity, implying that parametric methods assuming normality may be inappropriate and that robust or non-parametric approaches should be preferred for subsequent analyses.

**Figure 1:**
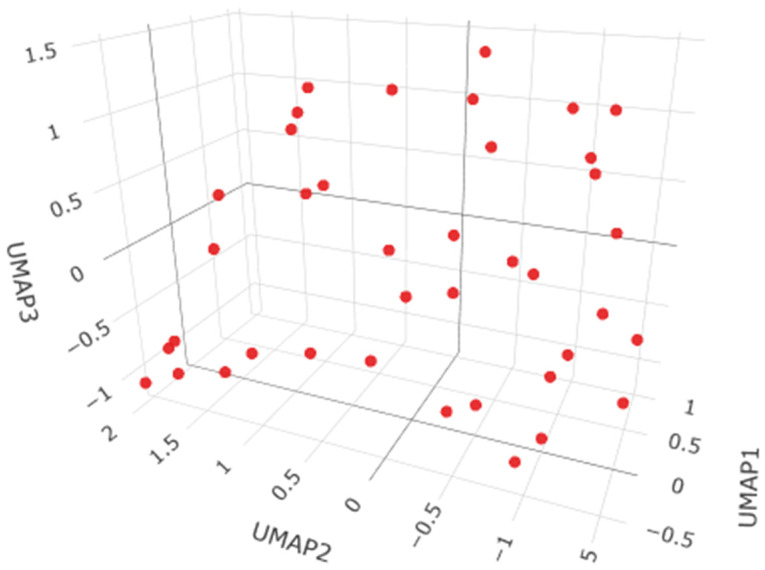
The HDBSCAN clustering analysis for the brain samples.

The HDBSCAN clustering analysis performed on 39 observations (minPts = 5) did not identify any meaningful clusters, classifying all objects as noise (Figure 1). This result indicates the absence of well-defined density-based groupings in the data, suggesting that the observations do not exhibit a natural cluster structure and instead form a largely homogeneous or continuously distributed set without distinct subpopulations.

**Figure 2:**
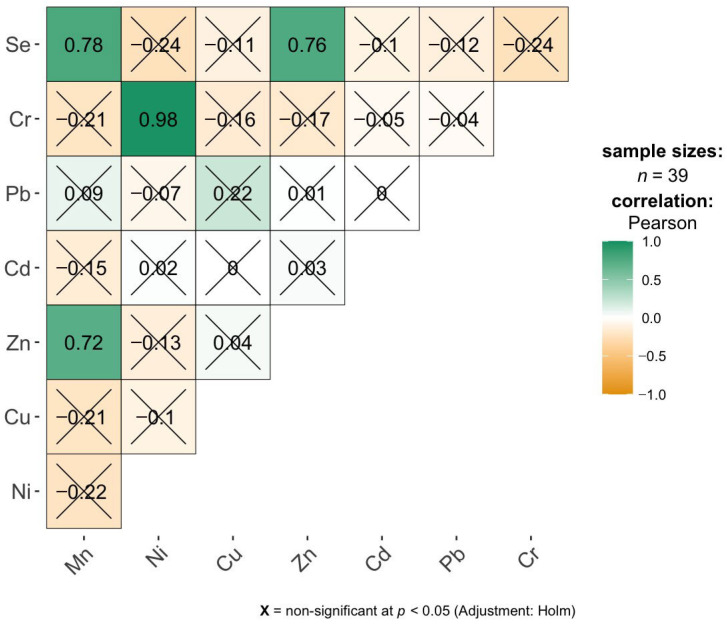
Pearson correlation analysis of brain element concentrations.

The correlation matrix for the brain samples indicates a very limited number of statistically significant linear relationships between elemental concentrations (Figure 2). After applying the Holm correction for multiple comparisons, only three positive correlations remain significant: a very strong correlation between Cr and Ni (r = 0.98), and high correlations between Se and Mn (r = 0.78) as well as Se and Zn (r = 0.76). This suggests that, in the studied group, Cr and Ni concentrations increase almost proportionally, while Se shows a clear co-variation with both Mn and Zn. All other element pairs do not exhibit significant correlations after adjustment, with correlation coefficients generally low and close to zero, indicating weak or negligible linear associations. Overall, the results imply that elemental profiles in brain tissue are largely independent, with only a few specific pairs potentially reflecting shared accumulation pathways, metabolic regulation, or common exposure sources.

### 2.2. Elemental Concentration in the Liver

In the liver samples, most elemental concentrations deviate significantly from normality according to the Shapiro–Wilk test with Holm correction (all adjusted *p*-values < 0.001), with the notable exception of Zn, for which no evidence against normality was found (*p* = 0.940). Strong positive skewness and very high kurtosis are observed for several elements, particularly Cu, Cd, Pb, and Cr, indicating highly right-skewed distributions with heavy tails and extreme values, as also reflected by large discrepancies between means and medians and very high maximum concentrations (e.g., Cu and Cd) (Table 2). In contrast, Mn exhibits an approximately symmetric distribution with near-zero skewness and kurtosis, yet still departs from normality, suggesting subtle but statistically detectable deviations. Overall, the liver data show pronounced heterogeneity and non-Gaussian structure for most elements, implying that parametric methods based on normality assumptions are generally inappropriate, with Zn being the only variable plausibly consistent with a normal distribution in this tissue.

**Figure 3:**
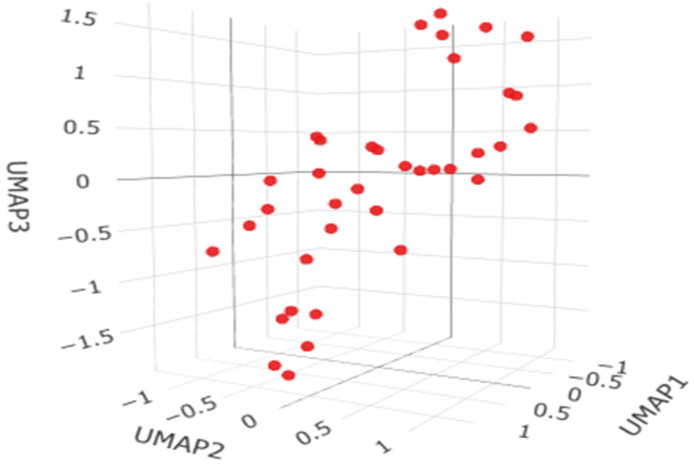
The HDBSCAN clustering analysis for liver samples.

The HDBSCAN clustering analysis for the liver dataset (37 observations, minPts = 5) did not identify any meaningful clusters, with all observations classified as noise (Figure 3). This outcome indicates that the data lack a clear density-based clustering structure and do not form distinct subgroups, suggesting a continuous or homogeneous distribution of observations rather than separable clusters.

**Figure 4:**
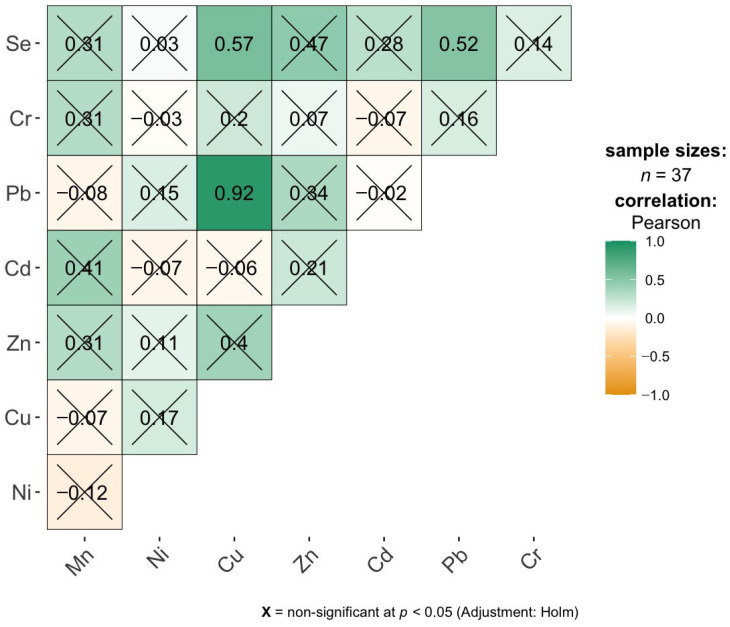
Pearson correlation analysis of liver element concentrations.

The correlation matrix for the liver samples reveals several moderate to strong positive associations between elemental concentrations at the descriptive level, although most of them do not remain statistically significant after applying the Holm correction for multiple testing (Figure 4). The strongest observed correlation is between Pb and Cu (r = 0.92), indicating a very high co-variation between Pb and Cu concentrations in liver tissue. Other relatively strong positive correlations include Se with Cu (r = 0.57), Se with Zn (r = 0.47), Se with Cd (r = 0.28), Se with Pb (r = 0.52), and Pb with Zn (r = 0.84), suggesting that Se and Pb tend to co-occur with several other elements. However, all these correlations are marked as non-significant after adjustment, implying that, despite their magnitude, they cannot be considered statistically robust given the sample size and multiple testing. Most remaining correlations are weak and close to zero. Overall, the liver data suggest the presence of some potentially meaningful co-accumulation patterns, particularly involving Pb, Cu, and Se, but these relationships should be interpreted cautiously and treated as exploratory rather than confirmatory.

### 2.3. Elemental Concentration in the Lungs

In the lung samples, most elemental concentrations show significant departures from normality based on the Shapiro–Wilk test with Holm correction (adjusted *p*-values ≤ 0.001 for all variables except Se). The distributions of Mn, Ni, Cd, Pb, and Cr are strongly right-skewed with high kurtosis, indicating pronounced asymmetry and heavy tails, consistent with the presence of extreme observations and substantial heterogeneity across subjects (Table 3). Cu and Zn also significantly deviate from normality despite exhibiting more moderate skewness and kurtosis, suggesting that even relatively symmetric-looking distributions fail to meet Gaussian assumptions. In contrast, Se does not significantly depart from normality (*p* = 0.128), although its skewness and kurtosis values still indicate some degree of asymmetry. Overall, the lung data are largely non-Gaussian, implying that robust or non-parametric statistical methods are generally more appropriate, with Se being the only element plausibly consistent with a normal distribution in this tissue.

**Figure 5:**
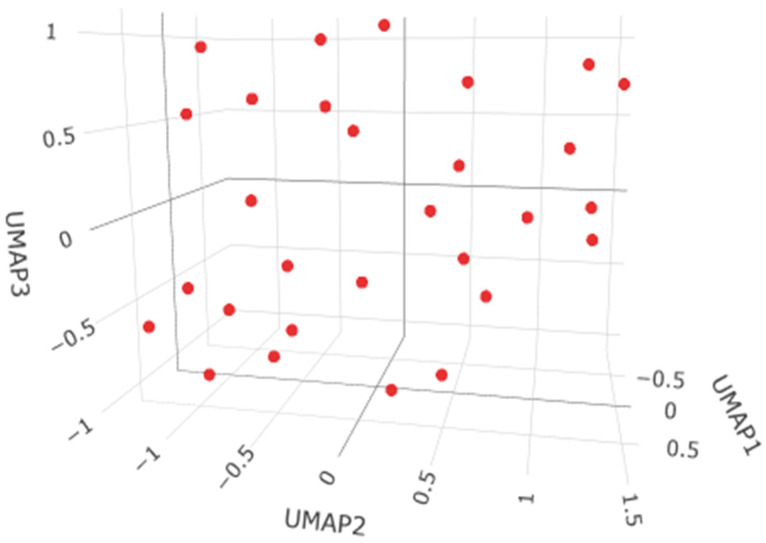
The HDBSCAN clustering analysis for the lungs samples.

The HDBSCAN clustering analysis for the lung dataset (29 observations, minPts = 5) did not reveal any distinct clusters, with all observations classified as noise (Figure 5). This result suggests that the data do not exhibit a clear density-based clustering structure and that the observations do not separate into well-defined subgroups, indicating a lack of natural clustering in the dataset.

**Figure 6:**
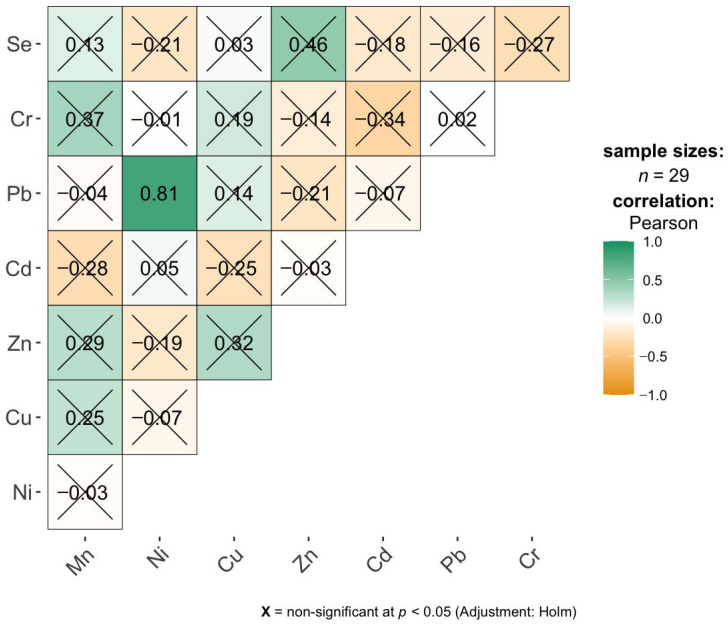
Pearson correlation analysis of lung element concentrations.

The correlation matrix for the lung samples shows only one statistically significant association after Holm correction, namely a strong positive correlation between Pb and Ni (r = 0.81), indicating a pronounced co-variation of Pb and Ni concentrations in lungs (Figure 6). This suggests that these two elements may share common exposure sources or similar accumulation mechanisms in the respiratory system. All other correlations are non-significant after adjustment and are generally weak to moderate in magnitude. Although some descriptive correlations appear relatively high (e.g., Zn with Cu, r = 0.82; Cr with Mn, r = 0.87; Se with Zn, r = 0.46), they do not reach statistical significance when controlling for multiple testing. Overall, the results indicate that elemental concentrations in lungs are largely independent, with Pb and Ni being the only pair showing a robust and statistically reliable linear relationship.

### 2.4. Elemental Concentration in the Bronchi

In the bronchi samples, most elemental concentrations also exhibit departures from normality, as indicated by the Shapiro–Wilk test with Holm correction, with significant results for Mn, Ni, Cu, Zn, Cd, and Pb (adjusted *p*-values ≤ 0.005). These variables show positive skewness and elevated kurtosis, particularly for Ni, Cu, Cd, and Cr, suggesting right-skewed distributions with heavy tails and the presence of extreme observations (Table 4). In contrast, Cr and Se do not significantly deviate from normality (*p* = 0.069 and *p* = 0.095, respectively), and their distributions are comparatively more symmetric, with low skewness and kurtosis close to zero. Overall, the bronchi data display substantial heterogeneity and asymmetry for most elements, although the degree of non-normality appears less extreme than in the brain for some variables (e.g., Mn and Zn), indicating that non-parametric or robust methods remain generally advisable, with possible exceptions for Cr and Se.

**Figure 7:**
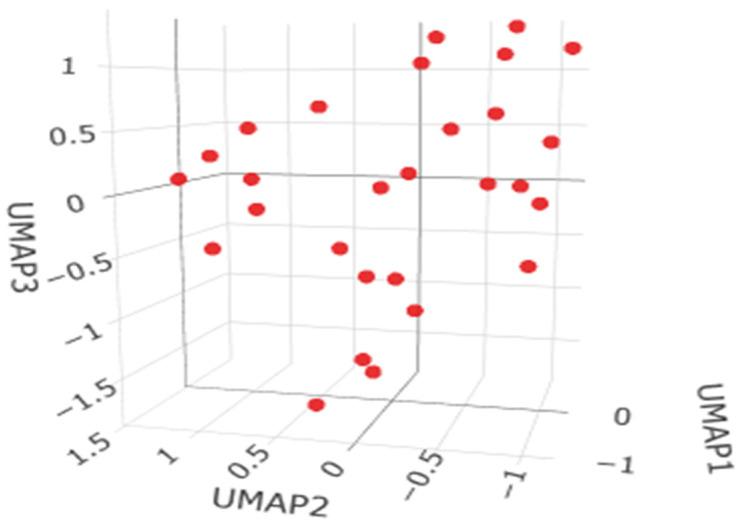
The HDBSCAN clustering analysis for the bronchi samples.

The HDBSCAN clustering analysis for the bronchi dataset (28 observations, minPts = 5) did not detect any distinct clusters, with all observations classified as noise (Figure 7). This indicates that the data do not exhibit a clear density-based clustering structure, suggesting the absence of well-defined subgroups and a lack of natural separation among the observations.

**Figure 8:**
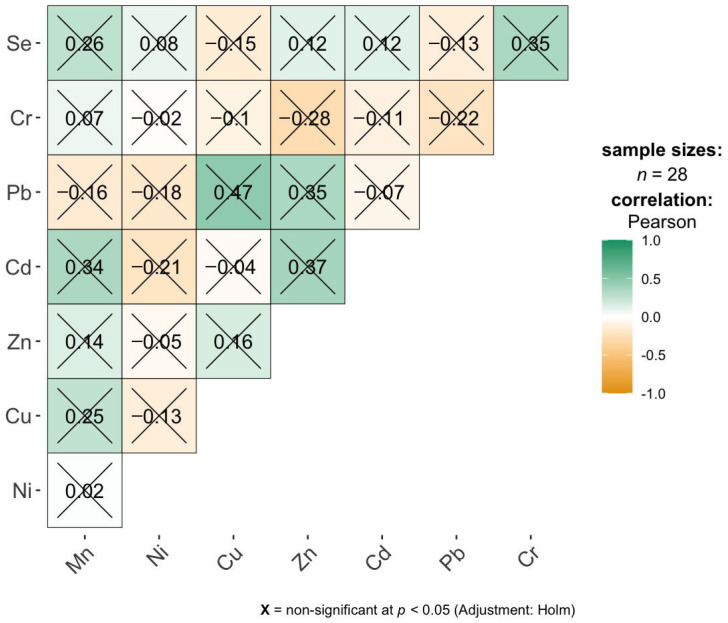
Pearson correlation analysis of bronchi element concentrations.

The correlation matrix for the bronchi samples shows only a small number of moderate to strong linear associations between elemental concentrations, and none of them remain statistically significant after applying the Holm correction for multiple testing (Figure 8). The strongest positive correlations are observed between Cd and Mn (r = 0.84), Cd and Zn (r = 0.87), Pb and Zn (r = 0.85), and Se and Cr (r = 0.85), indicating pronounced co-variation between these element pairs at the descriptive level. However, all correlations are marked as non-significant after adjustment, which suggests that these relationships are not sufficiently robust given the sample size and the number of comparisons. Most other pairs exhibit weak correlations close to zero, implying little or no linear association. Overall, the results indicate that elemental concentrations in bronchi do not form stable, statistically reliable correlation patterns, and the observed associations should be interpreted cautiously as exploratory rather than conclusive.

### 2.5. Comparison of the Elemental Composition of the Organs

Permutational Multivariate Analysis of Variance (PERMANOVA) is a semi-parametric method of determining significance based on dissimilarity measures that does not assume multivariate normality of the distribution. A permutational MANOVA (PERMANOVA) was used instead of a classical parametric MANOVA because the key assumptions required for the latter were clearly violated. The Mardia tests for multivariate normality indicated significant departures from normality in both skewness and kurtosis (*p* < 0.001), demonstrating that the joint distribution of variables is not multivariate Gaussian (Table 5).

In addition, Box’s M test showed a highly significant result (*p* < 0.001), indicating heterogeneity of covariance matrices across groups, thus violating the assumption of homoscedasticity (Table 6).

The assessment of homogeneity of variances across tissue groups indicates that this assumption is violated for several elements (Table 7).

Specifically, significant differences in dispersion between groups were observed for Cd (*p* = 0.0018), Mn (*p* < 10^−12^), Se (*p* = 0.0003), and Zn (*p* < 10^−5^), demonstrating that the variability of these elemental concentrations differs substantially across tissues. In contrast, no significant heterogeneity of variances was found for Cr, Cu, Ni, and Pb (*p* > 0.05), suggesting comparable dispersion for these elements across groups. Since parametric MANOVA is sensitive to both non-normality and unequal covariance structures, especially in moderate sample sizes, its results would be unreliable in this setting. PERMANOVA, being a non-parametric, permutation-based method, does not rely on distributional assumptions and is therefore more appropriate for these data, providing a robust framework for testing group differences under severe violations of classical MANOVA assumptions.

The PERMANOVA results indicate a statistically significant effect of tissue area on the multivariate elemental profile. The factor Area explains approximately 42.2% of the total variance in the data (R^2^ = 0.422), which represents a substantial proportion of the overall variability. The associated pseudo-F statistic is high (F = 31.40), and the permutation-based *p*-value is highly significant (*p* = 1 × 10^−4^), indicating that the observed group differences are very unlikely to have arisen by chance. These results provide strong evidence that the multivariate composition of elemental concentrations differs significantly between the analyzed tissue areas, with tissue type being a major determinant of the overall elemental profile.

Since the assumptions for parametric ANOVA tests were not met (lack of normality, heterogeneity of variance), the nonparametric Kruskal–Wallis test was used for each element separately, with Holm correction for multiple comparisons (Table 8).

The Kruskal–Wallis test results indicate statistically significant differences in elemental concentrations between tissue groups for almost all analyzed elements. After applying Holm correction for multiple comparisons, very strong group effects are observed for Mn, Zn, Cd, and Se (all adjusted *p*-values < 10^−15^), demonstrating highly significant differences in their distributions across the four tissues. These elements show the largest χ^2^ statistics, indicating pronounced and systematic variation between organs.

Moderate but still statistically significant group differences are found for Cr, Cu, and Pb (adjusted *p*-values ranging from 10^−6^ to 10^−3^), suggesting that their concentrations also vary significantly between tissues, although the magnitude of the effect is smaller compared to Mn, Zn, Cd, and Se. In contrast, no statistically significant differences are observed for Ni (*p* = 0.079 after correction), indicating that nickel concentrations do not differ reliably between tissue types.

For elements with significant differences in the Kruskal–Wallis test, post hoc analysis was performed using Dunn’s test with Holm correction (Table 9).

The pairwise Dunn post hoc comparisons reveal clear and element-specific patterns of differences between tissues.

**Figure 9:**
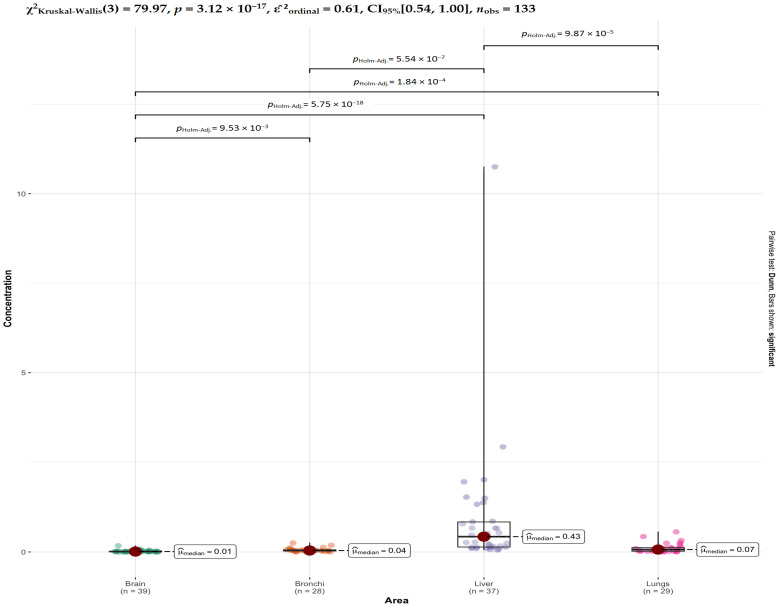
Comparative analysis of Cd concentrations across tissues. Results are presented as box-and-whisker plots (median, IQR, and range) with overlaid individual data points. Horizontal brackets indicate pairwise Dunn’s post hoc comparisons, with corresponding Holm-adjusted *p*-values displayed above. The global Kruskal–Wallis test result is provided as an overall summary of variance.

For Cd, highly significant differences are observed between the liver and all other tissues (brain, bronchi, and lungs), indicating that cadmium concentrations in the liver are markedly different from those in the remaining organs (Figure 9). Additional significant differences are found between brain and lungs and between brain and bronchi, whereas bronchi and lungs do not differ significantly, suggesting similar Cd levels in these two tissues.

For Cr, significant differences are mainly driven by comparisons involving the lungs (Figure S1). Both bronchi and liver differ strongly from lungs, and brain also differs from lungs, while no significant differences are observed among brain, bronchi, and liver. This indicates that chromium concentrations in lung tissue are distinct from the other organs, which show relatively comparable levels.

For Cu, significant differences are found between liver and both bronchi and lungs, as well as between brain and bronchi and brain and lungs (Figure S2). However, brain and liver do not differ significantly, and neither do bronchi and lungs. This suggests a gradient in copper concentrations, with liver and brain forming one group and bronchi and lungs forming another.

**Figure 10:**
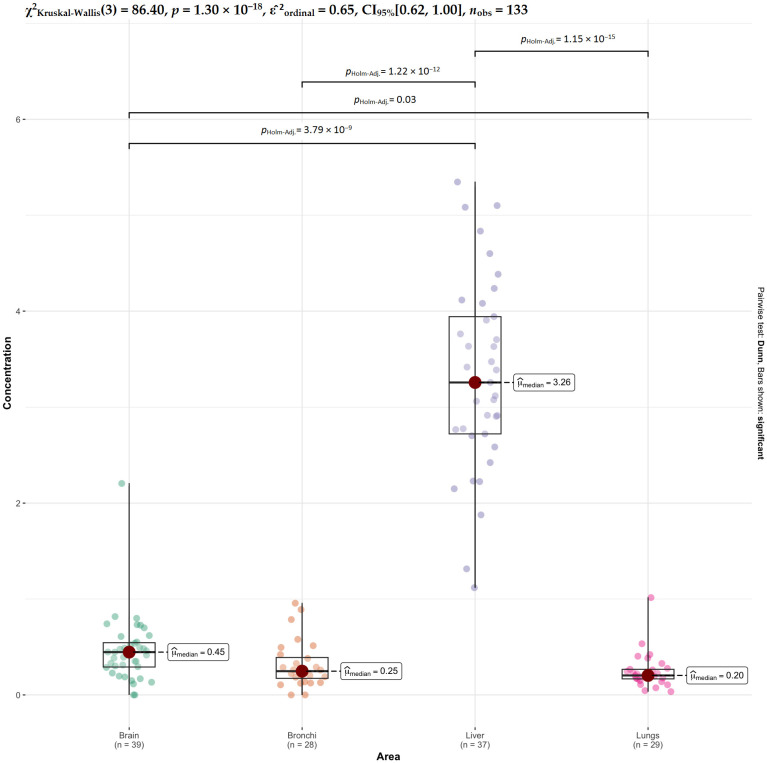
Comparative analysis of Mn concentrations across tissues.

For Mn, the strongest contrasts involve the liver, which differs highly significantly from all other tissues (Figure 10). Brain also differs from lungs, while no significant differences are observed between brain and bronchi or between bronchi and lungs. This indicates that manganese is particularly elevated or reduced in liver compared to the other organs, with relatively similar levels among respiratory tissues and brain.

For Ni, none of the pairwise comparisons are statistically significant after Holm correction (Figure S3). This confirms the Kruskal–Wallis result and indicates that nickel concentrations do not differ meaningfully between any of the examined tissues.

For Pb, significant differences are observed only between brain and liver and between liver and lungs, whereas all other pairwise comparisons are non-significant (Figure S4). This suggests that lead concentrations in the liver differ from those in brain and lungs, while bronchi do not differ significantly from any other tissue.

**Figure 11:**
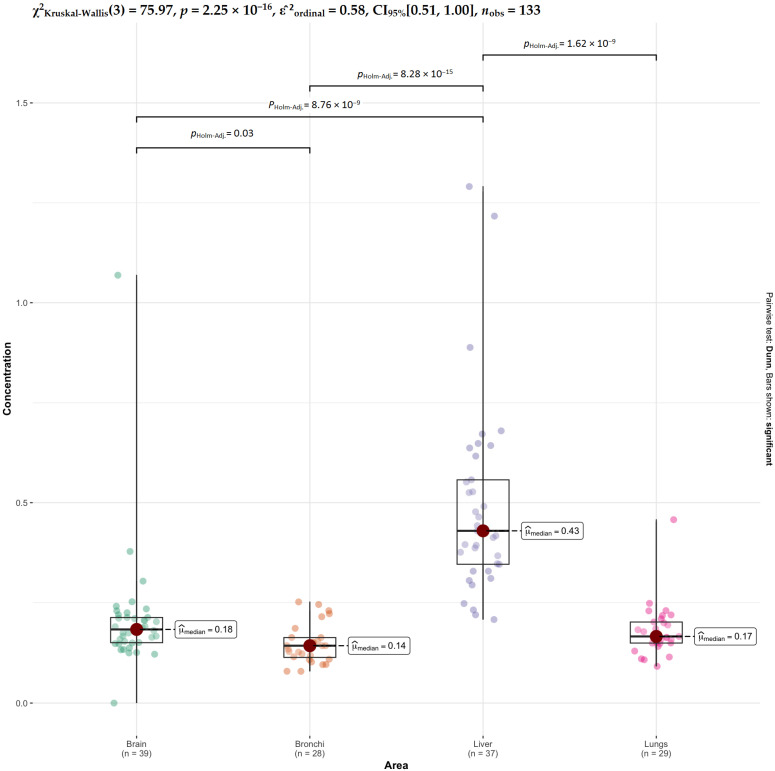
Comparative analysis of Se concentrations across tissues.

For Se, highly significant differences are again driven by the liver, which differs from bronchi, lungs, and brain (Figure 11). A weaker but significant difference is also observed between brain and bronchi, while no differences are found between brain and lungs or between bronchi and lungs. This indicates a strong liver-specific pattern for selenium, with relatively similar levels across the other tissues.

**Figure 12:**
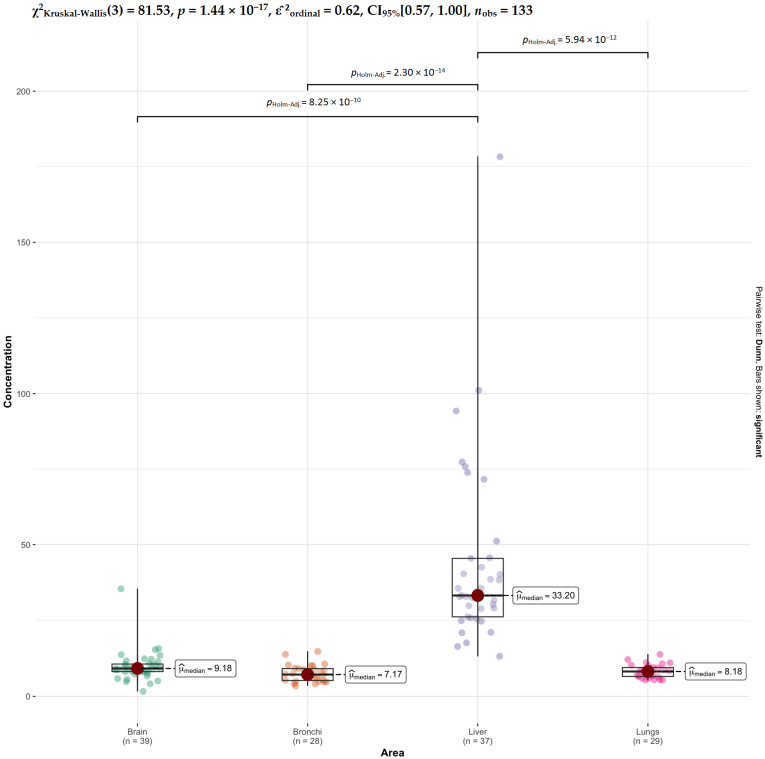
Comparative analysis of Zn concentrations across tissues.

Finally, for Zn, very strong differences are observed between liver and all other tissues (brain, bronchi, and lungs), whereas no significant differences are found among brain, bronchi, and lungs (Figure 12). This demonstrates that zinc concentrations are distinctly different in liver tissue, while the remaining organs form a relatively homogeneous group.

Overall, these results show that the liver is the primary driver of tissue-specific differences for most elements (Cd, Mn, Se, Zn, and partly Cu and Pb), whereas the lungs show specific differences mainly for Cr. Nickel stands out as the only element with no detectable tissue-specific variation. This pattern indicates strong organ-specific accumulation mechanisms, particularly for the liver, which acts as the main site of differentiation in elemental concentrations.

### 2.6. Changes in Element Levels by BMI, Age, and Gender

The linear regression analyses performed separately for each tissue area indicate that none of the considered demographic or anthropometric predictors (BMI, age, and sex) are significantly associated with elemental concentrations after correction for multiple testing (Table 10). In the brain and bronchi tissues, all regression coefficients are small in magnitude and non-significant, with wide confidence intervals including zero, suggesting no detectable linear effects of BMI, age, or sex on elemental levels. Similarly, in the lungs, although some nominal associations are observed before correction (e.g., BMI with Cd and sex with Cd). Women have higher Cd concentrations in the lungs (mean = 0.1903 µg/g) than men (mean = 0.0899 µg/g). The Cd concentration in lung samples increases with BMI. However, none of these trends remain significant after Holm adjustment, indicating that these effects are not robust. In the liver, a few predictors show relatively larger effect sizes at the descriptive level, particularly BMI for Cu and sex for Zn. Women have higher Zn in liver (mean = 57.6929 µg/g) in comparison to men (mean = 40.5753 µg/g), and the Cu level in liver increases with the increasing BMI values. However, these associations also lose significance after multiple testing correction. Overall, the results consistently show that inter-individual variability in elemental concentrations within each tissue cannot be explained by BMI, age, or sex in a statistically reliable way, suggesting that other biological, environmental, or exposure-related factors play a more dominant role in determining elemental accumulation patterns in these organs.

## 3. Discussion

Across all analyzed tissues (brain, bronchi, liver, and lungs), the HDBSCAN clustering procedure consistently failed to identify any meaningful cluster structure. For each dataset, using the same parameter setting (minPts = 5), all observations were classified as noise and zero clusters were detected. This uniform outcome indicates that the multivariate profiles of elemental concentrations do not form well-defined density-based groupings in any of the examined organs. Instead, the data appear to be continuously distributed, without clear separation into distinct subpopulations, suggesting substantial overlap between individuals and the absence of natural clusters in the studied biological samples.

Overall, these results provide strong evidence that tissue type has a substantial impact on the distribution of most elemental concentrations, with only Ni showing no significant tissue-specific variation. This supports the conclusion that organ-specific accumulation patterns exist for the majority of analyzed elements.

### 3.1. Hepatic Sequestration of Toxic Metals and Essential Trace Elements

In our study, we observed preferential accumulation of Cd and Pb in liver tissue. This is supported by numerous toxicological studies of the liver, which, as a major metabolic organ, is one of the first sites reached by toxic metals after absorption [26]. IARC (International Agency for Research on Cancer) (e.g., volumes 58 and 100 C) classifies Cd as a Group 1 carcinogen and describes its accumulation in parenchymal organs, including the liver [27]. The ATSDR (Agency for Toxic Substances and Disease Registry) document: Toxicological Profile of Cadmium describes in detail the kinetics of Cd, indicating that approximately 50–75% of the total body Cd content is located in the liver and kidneys [28]. Other sources also report that Cd accumulates in the liver (average about 4 mg in an adult) and its half-life ranges from 10 to 30 years [29,30]. Luis E. Gomez-Quiroz et al. identify the liver as the “primary target organ” of Cd toxicity, both in acute and chronic exposure [31]. This study, based on data from the National Health and Nutrition Examination Survey, notes a significant correlation between Cd levels and liver enzyme damage (ALT, AST, GGT). The liver is considered as one of the main reservoir of lead in the human body, holding 33% of the lead content [32,33]. However, there are important differences in the way these metals are stored: while Cd binds to sulfhydryl groups (−SH) in metallothioneins, it is only temporarily stored in soft tissues [34], whereas Pb toxicity results from Ca and Zn substitution and the generation of oxidative stress [15,35,36,37,38,39,40]. In our study, a strong correlation was observed between Pb levels and Cu, Se, and Zn, suggesting similar co-accumulation patterns.

The observed preferential accumulation of essential trace elements, such as zinc (Zn), selenium (Se), manganese (Mn), and copper (Cu), in the liver is due to its function as a metabolic center [21]. The liver requires vast amounts of these elements as cofactors for thousands of enzymes: Zn is essential for over 300 enzymes, including DNA polymerases (liver regeneration) [41]; Cu and Mn are essential for superoxide dismutase (SOD), which neutralizes free radicals produced during drug and alcohol detoxification [21,42]; and Se is a component of glutathione peroxidase, a major antioxidant that protects the liver from damage [43]. Micronutrients absorbed in the intestines are stored in the liver after binding to transport and storage proteins [44]. Metallothioneins (MT) are the most important storage proteins for Zn and Cu [45]. Ceruloplasmin is a protein in the liver that enables the transport of Cu to other tissues [42]. The liver also synthesizes selenoprotein P, responsible for the transport of Se to the brain and kidneys [43]. The liver possesses Cu and Mn transporters (ABC transporters) and ATPases, such as ATP7B. For many metals (especially Cu and Mn), the liver is not only a storage site but also a site for the removal of excess Cu from the body. Most Cu and Mn are eliminated from the body through bile secretion [42,46]. If this process is disturbed (e.g., in the case of Cu in Wilson’s disease), pathological liver overload occurs, leading to cirrhosis [47].

Iritas et al. [10] determined the concentrations of toxic metals (cadmium and lead) and trace elements (zinc and copper) in post-mortem liver tissue samples from residents of Ankara, Turkey. The element levels reported in their study were several times higher than those observed in our current research. This discrepancy may stem not only from actual regional differences but also from methodological variations; specifically, the Ankara group performed their analysis on dried tissue samples. Copper and zinc levels were determined using Flame Atomic Absorption Spectrometry (FAAS), while lead and cadmium were measured using Graphite Furnace Atomic Absorption Spectrometry (GFAAS). The mean concentrations reported were 29 µg/g for Cu, 216 µg/g for Zn, 0.39 µg/g for Pb, and 4.38 µg/g for Cd. Despite the differences in absolute values, the proportions between trace elements remained consistent across both studies, with a Zn/Cu ratio of approximately 7. In contrast, the Cd/Pb ratio in the Turkish study was nearly twice as high as ours, which may reflect differences in environmental exposure between the populations. Similarly, a Spanish study by García et al. [16], involving residents living near the Constantí Hazardous Waste Incinerator (HWI), reported trace element concentrations in autopsy liver tissues comparable to our findings. They observed levels of 0.76 µg/g for Cd and 0.23 µg/g for Pb, whereas our study yielded 0.891 µg/g for Cd and 0.188µg/g for Pb. While levels of toxic and essential elements are widely investigated globally and correlated with demographic data or pathologies, few studies emphasize inter-element correlations or concentration ratios. Such parameters are crucial as they may indicate shared bioaccumulation pathways, ionic mimicry, or disruptions in elemental homeostasis. Similar imbalances have been documented in patients with acquired hepatocerebral degeneration (AHD) [48]. These authors reported elevated levels of Mn, Li, B, Ni, As, Sr, Mo, Cd, Sb, Tl, and Pb, along with an increased Cu/Se ratio, while Se and Rb levels were decreased in patients with hepatic dysfunction. Notably, their findings showed that elevated Pb levels coexist with increased Cu concentrations, a trend that aligns with the results obtained in our study.

### 3.2. Pulmonary Deposition and Long-Term Sequestration of Inhaled Chromium

The observed preferential accumulation of Cr in the lungs, which is confirmed by epidemiological and toxicological studies, results from the fact that the main route of exposure to Cr is the respiratory tract and inhalation of dust and fumes, and additionally, the specific chemical nature of chromium hinders its removal from lung tissue [49,50]. Chromium compounds, especially low-solubility chromates, are deposited directly on the bronchial epithelium and alveoli [49]. Alveolar macrophages absorb them but are often unable to neutralize or effectively remove them, leading to their long-term retention in lung tissue [51,52]. The key to understanding the accumulation is the oxidation state of chromium. Hexavalent chromium (Cr(VI)) is highly soluble and readily crosses cell membranes via sulfate transporters, a process known as “molecular mimicry” [51,52]. When Cr(VI) enters a lung cell, it is reduced to trivalent chromium (Cr(III)), which is much less mobile and has a high affinity for proteins and nucleic acids [51,52]. Cr(III) formed inside the lung cell becomes “trapped” there by binding to macromolecules, which prevents its excretion into the bloodstream [52]. Post-mortem studies of tissues from occupationally exposed individuals show that Cr concentrations in the lungs can be 10 to 100 times higher than in other organs such as the liver or kidneys [50,53]. Studies of workers exposed to chromate document that Cr can remain in the lungs for decades after exposure has ceased; for example, Hirose et al. (2002) reported stable Cr-containing deposits in the fibrous tissue of the lungs [53]. The International Agency for Research on Cancer (IARC) classifies Cr(VI) compounds as carcinogenic (Group 1) precisely because of their specific accumulation and ability to induce lung cancer, mainly squamous cell carcinoma and adenocarcinoma [54]. Recent analyses by Meaza et al. analyze Cr(IV) prolonged accumulation in the bronchial epithelium [49]. The ATSDR toxicological profile for Cr confirms that the lungs are the main target organ after inhalation [28].

dos Santos et al. [55] investigated the concentrations of heavy metals and polonium-210 (^210^Po) in autopsy lung tissues from residents of São Paulo, Brazil. Compared to our findings, where Cr concentrations ranged from 0.1 to 0.3 μg/g, the residents of São Paulo exhibited significantly higher lung Cr levels, ranging between 0.7 and 3.7 μg/g. In another study, Morton et al. [56] analyzed multi-elemental profiles in cancerous lung tissue samples using inductively coupled plasma mass spectrometry (ICP-MS), reporting Cr concentrations in the range of 0.092–51.143 μg/g (median: 0.48 μg/g). Furthermore, Marmor et al. [57] examined metal concentrations in autopsy lung tissues collected between 2007 and 2011 from 76 individuals enrolled in the World Trade Center Health Registry (WTCHR) and 55 community controls. In their study, the median Cr concentration for both exposed and non-exposed individuals was 1.3 μg/g, which further highlights the variability of chromium accumulation in pulmonary tissues across different populations and environmental conditions. These comparisons suggest that the relatively low level of industrialization in south-eastern Poland does not pose a significant risk of chromium bioaccumulation in the local population. Our findings indicate that Cr levels in this region remain substantially lower than those recorded in highly urbanized or industrially burdened areas, such as São Paulo or the New York metropolitan area.

### 3.3. Inter-Elemental Correlations and Synergistic Accumulation of Metals in Studied Tissues

The existence of positive correlations between the concentrations of various elements in tissues (liver, lungs, brain, and bronchi) can be explained by three main mechanisms: a common exposure pathway, biological synergy, and the body’s defense mechanisms [21,26]. The positive correlations between the essential elements Se-Zn and Zn-Mn, observed in the brain, and Se-Cu in the liver, are primarily due to their homeostatic and antioxidant functions. Regarding the Common Response to Oxidative Stress: Zinc (Zn), copper (Cu), manganese (Mn), and selenium (Se) are components of key antioxidant enzymes, such as Cu/Zn-SOD, Mn-SOD, and glutathione peroxidase [7]. When the body is subjected to stress, the liver increases the retention of these elements to intensify the production of defensive enzymes [7,21]. Common Transport Proteins also play a role. Zn and Cu are coregulated by metallothioneins (MT) [58]. An increase in the concentration of one of them often induces MT synthesis, which in turn increases the tissue’s ability to bind the other element [58,59]. Furthermore, dietary sources contribute to these correlations, as these elements often occur together in the same foods, such as nuts, seeds, and meat, leading to their parallel delivery to the body [2].

In turn, the observed toxic and mixed correlations, such as Cr-Ni (brain), Pb-Cu and Pb-Se (liver), and Pb-Ni (lungs), result from industrial associations or detoxification attempts [26]. The Cr-Ni correlation is one of the strongest in tissue studies, linked by common emissions: Cr and Ni are the main components of stainless steel. In industrial processes like welding and galvanization, they almost always occur as companions in fumes and dust [49]. They are characterized by similar kinetics; both metals enter lung cells via similar pathways and tend to accumulate in alveolar macrophages [49]. Regarding Pb correlations (Pb-Cu, Pb-Ni, Pb-Se), it is known that Pb often disrupts trace element metabolism [60]. This positive correlation may result from lead damaging the excretory mechanisms of the liver and kidneys, leading to secondary, pathological accumulation of Cu and Ni, which the body is unable to efficiently eliminate [61,62]. The Pb-Se correlation is a classic example of detoxification [63]. Se has the ability to bind heavy metals by forming metal selenides. As Pb levels rise, the body “mobilizes” Se or increases its retention in tissues to neutralize lead toxicity [64]. Stable Pb-Se complexes are formed and deposited in tissues, revealing a positive correlation between both elements in chemical studies [63,65]. Another mechanism responsible for positive correlations is shared transporters (molecular mimicry). Many metals “masquerade” as essential trace elements, using the same “door” to the cell [66]. An example is the DMT1 (Divalent Metal Transporter 1), which transports Fe^2+^, Zn^2+^, and Mn^2+^, but also Pb^2+^ and Cd^2+^ [66]. High activity of this transporter during deficiency states, such as iron deficiency, causes the simultaneous absorption of the entire metal group into the tissue, creating positive correlations between them [66].

### 3.4. Influence of Sex and BMI on the Accumulation Profiles of Heavy Metals and Essential Elements in Target Organs

The study found that women have higher Cd concentrations in their lungs compared to men, with levels more than twice as high in women. This disparity may be linked to Fe metabolism [67]. It has also been hypothesized that sexual dimorphism in lung morphology, specifically the smaller pulmonary surface area in women, may alter the toxicokinetics of inhaled pollutants. Such anatomical constraints might contribute to increased retention times and elevated internal doses of toxins, leading to more persistent exposure than observed in men [68]. Women are more prone to low ferritin levels (which indicate their Fe stores) due to menstrual cycles. In an effort to absorb more Fe from their environment, the body may inadvertently absorb more Cd, as both metals utilize the same protein transporter, divalent metal transporter 1 (DMT1) and metal transporter protein 1 (MTP1) [69].

Additionally, women’s metabolism of xenobiotics and the higher density of receptors in their tissues may contribute to greater accumulation of pollutants in the lung alveoli [69]. Furthermore, the observation that Cd concentrations increase with body mass index (BMI) suggests that adipose tissue may act as a “storage” site for this metal or influence its retention in the body [70].

Cd is not a typical fat-soluble metal, but a higher Body Mass Index (BMI) is often linked to overall body inflammation and metabolic changes. Increased body weight can indicate greater environmental exposure—such as consuming more food or requiring more energy—or slower detoxification processes [71]. Additionally, individuals with a higher BMI tend to experience elevated levels of oxidative stress in the lungs, which can hinder the natural mechanisms that clear particulate matter containing heavy metals from the respiratory tract [72].

In terms of the liver, variations in element levels may be influenced by sex hormones and the liver’s role in energy storage. For instance, higher levels of Zn in women are associated with estrogen’s effects [73]. Estrogen promotes the production of metallothioneins in the liver—specialized proteins whose main function is to bind and store Zn and Cu. Because women generally have higher levels of estrogen, their livers produce more of these “depots,” enabling them to retain larger amounts of Zn [73].

Zn serves as a component of superoxide dismutase (Cu/Zn-SOD), an enzyme that helps protect against oxidative stress. Higher levels of Zn in women may act as a protective mechanism for liver tissue against metabolic damage [74]. Additionally, Cu levels in the liver tend to increase with a higher body mass index (BMI). While Cu is an essential nutrient, excessive amounts can become pro-oxidant, promoting the formation of free radicals. This correlation between Cu levels and BMI raises concerns about metabolic health 75, as a higher BMI is often linked to chronic, low-grade inflammation.

In response to inflammation, the liver increases the production of ceruloplasmin, a protein responsible for Cu transport. This results in greater retention of Cu within the liver [75]. There is also a growing risk of nonalcoholic fatty liver disease (NAFLD) as BMI increases. Research indicates that individuals who are overweight may experience impaired Cu export from the liver to the bile, causing copper to become “trapped” in liver tissue [76]. Cu plays a role in fat metabolism, and excessive accumulation due to high BMI may indicate that the mechanisms responsible for fatty acid combustion are overloaded. It is important to note that Zn and Cu are antagonistic—they compete for the same absorption sites and transport proteins [77].

## 4. Materials and Methods

### 4.1. Studied Population and Sample Characterization

Tissue samples were collected at the Department of Forensic Medicine, Medical University of Lublin. The study was approved by the Local Ethics Committee (Medical University of Lublin, Poland, approval number KE-0254/152/2021). Sample collection was approved by the Prosecutor’s Office responsible for the autopsies. According to Polish law, securing biological material during forensic autopsies requires the consent of the prosecutor supervising the proceedings. Such a decision is not dependent on obtaining additional consent from the family members of the deceased person. The study was conducted in accordance with the Code of Ethics of the World Medical Association and the Helsinki Declaration regarding human experimentation. Samples were collected from the subjects within 24 h of confirmation of death. The study included 45 individuals. Individuals who died in an advanced stage of decomposition and those who died in accidents with injuries to the studied organs were excluded from the study. The study population consisted of residents of a sparsely industrialized, agricultural region of southeastern Poland. The individuals died as a result of sudden death, or suicide. The demographic characteristics of the studied population are presented in Table 11.

Due to the exclusion of certain specimens, separate demographic and anthropometric summaries for each tissue type are presented in Table S1. Samples were excluded based on two primary criteria: (i) incomplete medical documentation, which prevented accurate correlation analysis, and (ii) insufficient sample mass (<0.5 g), which increased the risk of analytical error and could compromise the detection limits of the ICP-MS method. Detailed information regarding the sample size for each tissue type is provided in Table S1. This selective approach was adopted to ensure the highest data integrity and representativeness of the results.

### 4.2. Sample Collection Procedure

Tissue samples were collected by qualified pathologists. Samples weighing 0.5–3.3 g were rinsed with ultrapure water (Milli-Q, Millipore, Raleigh, NC, USA; resistivity: 18.2 MΩ cm), dried on a sterile filter paper, weighed on an analytical balance and stored in decontaminated polypropylene tubes at −80 °C until further analysis. Instruments (knives, forceps and scissors) used during autopsy were immersed in 10% HNO_3_ and then rinsed with ultrapure deionized water to minimize the likelihood of sample contamination.

### 4.3. Sample Preparation

Tissue samples were predigested with 7 mL of 69% Suprapur Nitric Acid 65% HNO3 (Baker, Radnor, PA, USA) and 1 mL of deionized water. Closed digestion was performed in closed Teflon containers using a microwave digester using a Mars 6 microwave system (CEM, Matthews, NC, USA). Microwave digestion was performed according to the program: 20 min at maximum temperature 185 °C, rise time 10 min, holding time 10 min. After digestion, the digests were quantitatively transferred to sterile Falcon polypropylene conical tubes with Plug-Seal caps and 1 mL of 35% HCl ultrapure for trace metal analysis (Baker, Radnor, PA, USA) was added to stabilize some elements (Se). The sample was then diluted to a total volume of 25.0 mL by ultrapure water (>18.2 MΩ cm at 25 °C) obtained in the Milli-Q purification system (Millipore, Darmstadt, Germany). A sample without tissue was used as a control. The tissue mineralization and analytical procedures were performed according to validated laboratory protocols, consistent with the methodologies described in previous studies [19,21,22], which served as the benchmark for comparing our findings. This consistency ensures the reliability and comparability of the trace element concentration data across different populations.

### 4.4. Measurements Using ICP-MS

All sample were analyzed using ICP MS (PQ MS Q, Analytik Jena, Jena, Germany), which is equipped with Integrated Collision-Reaction Cell (iCRC). All detailed operating parameters are placed in Supplementary Material (Table S2). Multiple certified reference materials (CRMs) were used to validate the ICP MS method (Table S3). The selected CRMs, representing a similar matrix, were used in quality control of the analysis process, e.g., DB001 (human hair), ERM BB 184 (bovine muscle), BCR 185R (bovine liver), achieving the acceptable recovery (80–120%) for most elements. For elements with uncertified content, the standard addition method was additionally used. All ICP-MS measurements results are collected in Table S4.

### 4.5. Statistical Analysis

Element concentrations measured by ICP–MS were first summarized using a com-prehensive set of descriptive statistics (mean, standard deviation, median, mini-mum/maximum, skewness, kurtosis) supplemented with the median absolute deviation (MAD) as a robust dispersion metric, which is particularly informative under heavy-tailed and right-skewed distributions typical of trace-element data. Normality of each elemental distribution within each tissue was evaluated using the Shapiro–Wilk test, and the resulting *p*-values were adjusted with the Holm (Holm–Bonferroni) procedure to control the family-wise error rate across multiple elements tested in parallel. This combination of robust descriptive summaries (median, MAD) with formal normality diagnostics is appropriate because elemental concentrations frequently include zeros, extreme values, and heterogeneous variability across subjects and organs, which can invalidate purely Gaussian-based inference and motivate the downstream use of nonparametric and permutation-based procedures.

To explore latent structure and co-variation patterns within each tissue, density-based clustering was performed using HDBSCAN (with minPts = 5), and linear asso-ciations between pairs of elements were quantified using Pearson correlation coefficients with Holm adjustment over the full correlation matrix. HDBSCAN is suitable here because it can detect clusters of varying density and can explicitly label observations as noise, which is useful in biomedical datasets where subpopulations may be weakly separated or absent; indeed, classifying all observations as noise provides evidence against discrete subgroups and supports interpreting variability as continuous rather than clustered. Correlation analysis is an appropriate exploratory tool for assessing potential shared exposure sources or joint accumulation mechanisms (e.g., co-variation of metals), while Holm correction is essential because the number of pairwise correlations grows rapidly with the number of elements, inflating false positives if unadjusted *p*-values were used.

For inferential comparisons of multielement profiles across tissues, multivariate assumptions were formally checked and found to be violated: Mardia’s tests indicated strong departures from multivariate normality (skewness and kurtosis), Box’s M test indicated heterogeneity of covariance matrices across tissue groups, and Levene’s tests showed element-specific heteroscedasticity for several analytes. Given these violations, a permutation-based PERMANOVA was used instead of parametric MANOVA to test whether overall multivariate elemental composition differs by tissue, because PERMANOVA does not rely on multivariate Gaussianity and is more robust under un-equal covariance structures. For element-wise tissue comparisons, the Kruskal–Wallis test was applied (again with Holm correction across elements) to accommodate non-normal, heteroscedastic distributions; when global differences were detected, Dunn’s post hoc pairwise procedure with Holm adjustment was used to localize which tissue pairs differed, matching the rank-based nature of Kruskal–Wallis. Finally, to assess whether inter-individual variability within each tissue could be explained by demographics and anthropometrics, tissue-stratified linear regressions were fitted for each element with BMI, age, and sex as predictors, and Holm adjustment was applied across the multiple regression tests to avoid over-interpreting nominal associations in a high-multiplicity setting.

## 5. Conclusions

Our analysis indicates that human tissues, including the liver, lungs, bronchi, and brain, are significant sites for the accumulation of essential trace elements (such as zinc, copper, selenium, and manganese) as well as toxic metals (including cadmium, lead, chromium, and nickel). This accumulation reflects the balance between the body’s homeostasis and various factors like diet, pollution, and occupational exposure. The population we studied displays a consistent elemental profile, suggesting that local environmental conditions—such as soil composition and air quality—play a crucial role in trace element accumulation.

We also found statistically significant organ-specific accumulation patterns for most of the elements analyzed. Research shows that essential trace elements, like zinc, selenium, manganese, and copper, preferentially accumulate in the liver, which functions as a metabolic hub. In contrast, toxic metals such as cadmium, and lead are associated with a “metabolic trap” mechanism, leading to their prolonged preferential retention (*p* < 0.0001). Our statistical analysis reveals strong positive correlations between the concentrations of essential elements, such as selenium-zinc and zinc-manganese in the brain, as well as selenium-copper in the liver. This indicates an integrated antioxidant response at play.

Furthermore, the correlations observed between toxic and essential metals, like lead-copper and lead-selenium in the liver, suggest the possibility of molecular mimicry, which may explain how toxic metals can imitate essential elements and lead to pathological accumulation and metabolic disorders.

In summary, monitoring elemental profiles in postmortem tissues is essential for assessing heavy metal burdens and understanding their role in chronic diseases.

## Figures and Tables

**Table 1:** Descriptive statistics for ICP-MS measurements of chemical elements [µg/g w.w.] in the brain samples.

Variable	Mean	SD	Median	MAD	Min	Max	Skewness	Kurtosis	*p*
Mn	0.459	0.356	0.446	0.225	0	2.205	3.122	14.881	0
Ni	0.024	0.087	0	0	0	0.541	5.797	34.989	0
Cu	3.676	4.002	2.683	3.977	0	14.191	1.138	0.455	0
Zn	9.833	5.101	9.178	2.012	1.63	35.399	3.353	16.662	0
Cd	0.019	0.03	0.011	0.01	0	0.173	4.026	19.489	0
Pb	0.116	0.272	0	0	0	1.282	3.441	12.243	0
Cr	0.101	0.379	0.031	0.028	0	2.39	6.102	37.758	0
Se	0.207	0.153	0.183	0.048	0	1.069	4.866	27.642	0

**Table 2:** Descriptive statistics for ICP-MS measurements of chemical elements in the liver samples.

Variable	Mean	SD	Median	MAD	Min	Max	Skewness	Kurtosis	*p*
Mn	3.318	1.012	3.258	0.961	1.118	5.346	0.019	−0.207	0
Ni	0.042	0.105	0.008	0.012	0	0.525	3.758	14.663	0
Cu	6.128	16.192	2.4	3.07	0	99.96	5.703	33.756	0
Zn	43.715	31.029	33.199	11.321	13.197	178.293	2.688	9.208	0.94
Cd	0.891	1.794	0.425	0.431	0.055	10.75	4.896	26.768	0
Pb	0.188	0.406	0.085	0.074	0	2.418	4.925	26.662	0
Cr	0.054	0.168	0.017	0.015	0	1.01	5.486	31.467	0
Se	0.489	0.239	0.43	0.15	0.208	1.291	1.893	4.315	0

**Table 3:** Descriptive statistics for ICP-MS measurements of chemical elements in the lungs samples.

Variable	Mean	SD	Median	MAD	Min	Max	Skewness	Kurtosis	*p*
Mn	0.248	0.185	0.203	0.085	0.034	1.015	2.734	10.191	0
Ni	0.026	0.047	0.01	0.015	0	0.23	3.313	12.797	0
Cu	0.682	0.681	0.432	0.504	0	2.289	1.058	−0.056	0.001
Zn	8.199	2.191	8.184	2.436	5.362	13.808	0.7	−0.005	0
Cd	0.111	0.134	0.067	0.073	0	0.559	1.994	3.993	0
Pb	0.042	0.066	0.017	0.017	0	0.264	2.881	8.158	0
Cr	0.085	0.077	0.049	0.041	0.011	0.319	1.519	1.848	0
Se	0.18	0.066	0.166	0.042	0.091	0.457	2.627	10.557	0.128

**Table 4:** Descriptive statistics for ICP-MS measurements of chemical elements in the bronchi samples.

Variable	Mean	SD	Median	MAD	Min	Max	Skewness	Kurtosis	*p*
Mn	0.314	0.243	0.248	0.171	0	0.957	1.355	1.467	0
Ni	0.038	0.118	0.004	0.006	0	0.586	4.229	18.62	0
Cu	0.915	1.49	0.251	0.373	0	5.761	2.499	6.031	0
Zn	7.538	2.829	7.173	2.952	3.45	14.771	0.833	0.499	0.005
Cd	0.054	0.056	0.04	0.024	0	0.251	2.23	5.574	0
Pb	0.092	0.131	0.041	0.056	0	0.459	1.915	2.964	0
Cr	0.024	0.062	0.012	0.018	0	0.331	4.875	24.944	0.069
Se	0.147	0.048	0.143	0.038	0.079	0.252	0.802	−0.090	0.095

**Table 5:** Assessment of multivariate normality assumptions prior to PERMANOVA analysis.

Test	Statistic	*p*-Value	Method	MVN
Mardia Skewness	7790.153	<0.001	asymptotic	✗ Not normal
Mardia Kurtosis	164.207	<0.001	asymptotic	✗ Not normal

**Table 6:** Assessment of the homogeneity of multivariate dispersions using Box’s M test.

Statistic	*p*-Value	Parameter	Method
1414.482	1.90 × 10^−226^	108	Box’s M-test for Homogeneity of Covariance Matrices

**Table 7:** Levene’s test results for homogeneity of variance for individual elements across tissue groups.

Element	df1	df2	Statistic	*p*
Cd	3	129	5.29085	1.79 × 10^−3^
Cr	3	129	0.523405	6.67 × 10^−1^
Cu	3	129	1.951885	1.24 × 10^−1^
Mn	3	129	26.70444	1.68 × 10^−13^
Ni	3	129	0.335908	7.99 × 10^−1^
Pb	3	129	1.200164	3.12 × 10^−1^
Se	3	129	6.787415	2.76 × 10^−4^
Zn	3	129	9.809411	7.11 × 10^−6^

**Table 8:** Results of Kruskal–Wallis tests comparing elemental concentrations across groups, including Holm-Bonferroni *p*-value adjustment.

Element	χ^2^	*p*	df	*p* Adj. (Holm)
Mn	86.4	1.30 × 10^−18^	3	1.04 × 10^−17^
Zn	81.53	1.44 × 10^−17^	3	1.01 × 10^−16^
Cd	79.97	3.12 × 10^−17^	3	1.87 × 10^−16^
Se	75.97	2.25 × 10^−16^	3	1.12 × 10^−15^
Cr	30.95	8.71 × 10^−7^	3	3.48 × 10^−6^
Cu	21.83	7.07 × 10^−5^	3	2.12 × 10^−4^
Pb	17.06	6.88 × 10^−4^	3	1.38 × 10^−3^
Ni	6.77	7.96 × 10^−2^	3	7.96 × 10^−2^

**Table 9:** Post hoc Dunn’s test results for pairwise comparisons of elemental concentrations between different tissues. Statistical significance is indicated as follows: **** *p* < 0.0001, *** *p* < 0.001, ** *p* < 0.01, * *p* < 0.05, and ns = non-significant (*p* > 0.05). All *p*-values were adjusted for multiple comparisons using the Holm-Bonferroni method.

Element	Group 1	Group 2	Z	*p* (Holm)	Significance
Cd	Brain	Liver	8.84	5.75 × 10^−18^	****
Cd	Brain	Lungs	4.01	1.84 × 10^−4^	***
Cd	Brain	Bronchi	2.82	9.53 × 10^−3^	**
Cd	Liver	Lungs	−4.22	9.87 × 10^−5^	****
Cd	Bronchi	Liver	5.31	5.54 × 10^−7^	****
Cd	Bronchi	Lungs	1.07	2.85 × 10^−1^	ns
Cr	Liver	Lungs	4.25	1.05 × 10^−4^	***
Cr	Brain	Lungs	2.89	1.54 × 10^−2^	*
Cr	Brain	Bronchi	−2.74	1.84 × 10^−2^	*
Cr	Brain	Liver	−1.51	2.62 × 10^−1^	ns
Cr	Bronchi	Lungs	5.24	9.82 × 10^−7^	****
Cr	Bronchi	Liver	1.33	2.62 × 10^−1^	ns
Cu	Liver	Lungs	−3.66	1.27 × 10^−3^	**
Cu	Brain	Bronchi	−2.82	1.92 × 10^−2^	*
Cu	Brain	Lungs	−2.76	1.92 × 10^−2^	*
Cu	Brain	Liver	1	6.35 × 10^−1^	ns
Cu	Bronchi	Lungs	0.08	9.38 × 10^−1^	ns
Cu	Bronchi	Liver	3.7	1.27 × 10^−3^	**
Mn	Liver	Lungs	−8.23	1.15 × 10^−15^	****
Mn	Brain	Liver	6.12	3.79 × 10^−9^	****
Mn	Brain	Lungs	−2.60	2.84 × 10^−2^	*
Mn	Brain	Bronchi	−1.74	1.65 × 10^−1^	ns
Mn	Bronchi	Lungs	−0.78	4.36 × 10^−1^	ns
Mn	Bronchi	Liver	7.32	1.22 × 10^−12^	****
Ni	Brain	Lungs	2.38	1.03 × 10^−1^	ns
Ni	Brain	Liver	2.01	2.22 × 10^−1^	ns
Ni	Brain	Bronchi	1.38	6.73 × 10^−1^	ns
Ni	Bronchi	Liver	0.48	1	ns
Ni	Bronchi	Lungs	0.92	1	ns
Ni	Liver	Lungs	0.5	1	ns
Pb	Brain	Liver	3.94	4.82 × 10^−4^	***
Pb	Brain	Lungs	0.81	5.55 × 10^−1^	ns
Pb	Brain	Bronchi	1.97	1.97 × 10^−1^	ns
Pb	Bronchi	Liver	1.67	2.86 × 10^−1^	ns
Pb	Bronchi	Lungs	−1.09	5.55 × 10^−1^	ns
Pb	Liver	Lungs	−2.84	2.22 × 10^−2^	*
Se	Liver	Lungs	−6.29	1.62 × 10^−9^	****
Se	Brain	Liver	5.98	8.76 × 10^−9^	****
Se	Brain	Bronchi	−2.53	3.39 × 10^−2^	*
Se	Brain	Lungs	−0.76	4.48 × 10^−1^	ns
Se	Bronchi	Lungs	1.67	1.92 × 10^−1^	ns
Zn	Bronchi	Lungs	0.78	4.36 × 10^−1^	ns
Zn	Bronchi	Liver	7.86	2.30 × 10^−14^	****
Zn	Liver	Lungs	−7.11	5.94 × 10^−12^	****
Zn	Brain	Liver	6.36	8.25 × 10^−10^	****
Zn	Brain	Bronchi	−2.06	1.18 × 10^−1^	ns
Zn	Brain	Lungs	−1.24	4.31 × 10^−1^	ns

**Table 10:** Regression coefficients and significance levels for the association between biological factors (BMI, Age, Sex) and tissue-specific elemental levels. Abbreviation: ns means non-significant.

Element	Predictor	β	95% CI Low	95% CI High	*p*	*p* (Holm)	Significance
Brain							
Mn	BMI	−0.0101	−0.0285	0.0083	2.69 × 10^−1^	1	ns
Mn	Age	−0.0009	−0.0062	0.0044	7.27 × 10^−1^	1	ns
Mn	SexM	−0.0494	−0.2265	0.1277	5.72 × 10^−1^	1	ns
Ni	BMI	−0.0019	−0.0106	0.0068	6.54 × 10^−1^	1	ns
Ni	Age	0.0007	−0.0018	0.0032	5.91 × 10^−1^	1	ns
Ni	SexM	0.0253	−0.0582	0.1088	5.39 × 10^−1^	1	ns
Cu	BMI	−0.1186	−0.4726	0.2355	4.97 × 10^−1^	1	ns
Cu	Age	0.0656	−0.0367	0.1679	1.99 × 10^−1^	1	ns
Cu	SexM	−0.4903	−3.8964	2.9157	7.70 × 10^−1^	1	ns
Zn	BMI	−0.0397	−0.3031	0.2236	7.59 × 10^−1^	1	ns
Zn	Age	−0.0173	−0.0934	0.0588	6.44 × 10^−1^	1	ns
Zn	SexM	−1.2957	−3.8289	1.2374	3.03 × 10^−1^	1	ns
Cd	BMI	0.0007	−0.0007	0.002	3.24 × 10^−1^	1	ns
Cd	Age	0.0002	−0.0002	0.0006	3.90 × 10^−1^	1	ns
Cd	SexM	0.0014	−0.0117	0.0145	8.32 × 10^−1^	1	ns
Pb	BMI	−0.0177	−0.0437	0.0082	1.72 × 10^−1^	1	ns
Pb	Age	0.0046	−0.0029	0.0121	2.16 × 10^−1^	1	ns
Pb	SexM	−0.0376	−0.2875	0.2122	7.59 × 10^−1^	1	ns
Cr	BMI	−0.0026	−0.0407	0.0354	8.89 × 10^−1^	1	ns
Cr	Age	0.003	−0.0080	0.014	5.78 × 10^−1^	1	ns
Cr	SexM	0.1096	−0.2563	0.4756	5.43 × 10^−1^	1	ns
Se	BMI	0.001	−0.0036	0.0057	6.45 × 10^−1^	1	ns
Se	Age	−0.0003	−0.0016	0.001	6.49 × 10^−1^	1	ns
Se	SexM	−0.0201	−0.0644	0.0242	3.60 × 10^−1^	1	ns
Bronchi							
Mn	BMI	0.0017	−0.0168	0.0201	8.49 × 10^−1^	1	ns
Mn	Age	0.0038	−0.0018	0.0094	1.74 × 10^−1^	1	ns
Mn	SexM	−0.0780	−0.2610	0.105	3.82 × 10^−1^	1	ns
Ni	BMI	0.0014	−0.0123	0.0152	8.28 × 10^−1^	1	ns
Ni	Age	0.0001	−0.0041	0.0043	9.62 × 10^−1^	1	ns
Ni	SexM	0.044	−0.0922	0.1802	5.06 × 10^−1^	1	ns
Cu	BMI	−0.0257	−0.1565	0.1051	6.85 × 10^−1^	1	ns
Cu	Age	0.0058	−0.0341	0.0457	7.63 × 10^−1^	1	ns
Cu	SexM	0.2901	−1.0065	1.5867	6.44 × 10^−1^	1	ns
Zn	BMI	0.0636	−0.2502	0.3773	6.75 × 10^−1^	1	ns
Zn	Age	−0.0137	−0.1093	0.082	7.68 × 10^−1^	1	ns
Zn	SexM	−0.1857	−3.2959	2.9246	9.02 × 10^−1^	1	ns
Cd	BMI	0.0024	−0.0019	0.0067	2.58 × 10^−1^	1	ns
Cd	Age	0.0000	−0.0014	0.0013	9.51 × 10^−1^	1	ns
Cd	SexM	−0.0208	−0.0637	0.022	3.20 × 10^−1^	1	ns
Pb	BMI	0.0021	−0.0070	0.0112	6.33 × 10^−1^	1	ns
Pb	Age	−0.0004	−0.0031	0.0024	7.89 × 10^−1^	1	ns
Pb	SexM	0.0185	−0.0717	0.1086	6.72 × 10^−1^	1	ns
Cr	BMI	−0.0011	−0.0022	0.0001	6.89 × 10^−2^	1	ns
Cr	Age	0.0003	0.0000	0.0007	6.78 × 10^−2^	1	ns
Cr	SexM	−0.0050	−0.0164	0.0064	3.69 × 10^−1^	1	ns
Se	BMI	0.0009	−0.0038	0.0056	6.97 × 10^−1^	1	ns
Se	Age	0.0003	−0.0011	0.0018	6.38 × 10^−1^	1	ns
Se	SexM	−0.0357	−0.0823	0.0109	1.25 × 10^−1^	1	ns
Liver							
Mn	BMI	0.0228	−0.0575	0.103	5.65 × 10^−1^	1	ns
Mn	Age	−0.0152	−0.0384	0.008	1.91 × 10^−1^	1	ns
Mn	SexM	0.2114	−0.5652	0.988	5.80 × 10^−1^	1	ns
Ni	BMI	−0.0030	−0.0115	0.0055	4.73 × 10^−1^	1	ns
Ni	Age	0.0006	−0.0019	0.003	6.47 × 10^−1^	1	ns
Ni	SexM	−0.0416	−0.1241	0.0408	3.08 × 10^−1^	1	ns
Cu	BMI	0.3768	0.1151	0.6385	6.57 × 10^−3^	6.17 × 10^−1^	ns
Cu	Age	0.0194	−0.0563	0.0951	6.02 × 10^−1^	1	ns
Cu	SexM	−2.1109	−4.6435	0.4217	9.84 × 10^−2^	1	ns
Zn	BMI	2.192	−0.1547	4.5386	6.58 × 10^−2^	1	ns
Zn	Age	0.5068	−0.1721	1.1856	1.37 × 10^−1^	1	ns
Zn	SexM	−33.1404	−55.8489	−10.4318	5.96 × 10^−3^	5.66 × 10^−1^	ns
Cd	BMI	0.0229	−0.1568	0.2026	7.95 × 10^−1^	1	ns
Cd	Age	0.0045	−0.0475	0.0564	8.61 × 10^−1^	1	ns
Cd	SexM	0.295	−1.4438	2.0338	7.30 × 10^−1^	1	ns
Pb	BMI	0.0083	−0.0057	0.0224	2.34 × 10^−1^	1	ns
Pb	Age	0.0016	−0.0025	0.0056	4.29 × 10^−1^	1	ns
Pb	SexM	0.0408	−0.0951	0.1766	5.42 × 10^−1^	1	ns
Cr	BMI	−0.0063	−0.0212	0.0087	3.95 × 10^−1^	1	ns
Cr	Age	−0.0028	−0.0071	0.0015	1.93 × 10^−1^	1	ns
Cr	SexM	−0.0663	−0.2108	0.0783	3.54 × 10^−1^	1	ns
Se	BMI	0.0112	−0.0014	0.0238	7.96 × 10^−2^	1	ns
Se	Age	0.0003	−0.0034	0.0039	8.76 × 10^−1^	1	ns
Se	SexM	0.0572	−0.0647	0.1791	3.43 × 10^−1^	1	ns
Lungs							
Mn	BMI	−0.0089	−0.0277	0.0098	3.31 × 10^−1^	1	ns
Mn	Age	−0.0022	−0.0080	0.0037	4.46 × 10^−1^	1	ns
Mn	SexM	−0.0753	−0.2721	0.1215	4.32 × 10^−1^	1	ns
Ni	BMI	0.0014	−0.0036	0.0064	5.69 × 10^−1^	1	ns
Ni	Age	−0.0012	−0.0028	0.0003	1.16 × 10^−1^	1	ns
Ni	SexM	0.0283	−0.0246	0.0812	2.76 × 10^−1^	1	ns
Cu	BMI	0.015	−0.0601	0.0901	6.80 × 10^−1^	1	ns
Cu	Age	0.0032	−0.0201	0.0264	7.78 × 10^−1^	1	ns
Cu	SexM	0.2454	−0.5425	1.0334	5.21 × 10^−1^	1	ns
Zn	BMI	−0.0034	−0.2170	0.2102	9.73 × 10^−1^	1	ns
Zn	Age	−0.0150	−0.0812	0.0512	6.40 × 10^−1^	1	ns
Zn	SexM	−1.7861	−4.0270	0.4547	1.11 × 10^−1^	1	ns
Cd	BMI	0.0118	0.0014	0.0222	2.82 × 10^−2^	1	ns
Cd	Age	0.0007	−0.0025	0.004	6.36 × 10^−1^	1	ns
Cd	SexM	−0.1621	−0.2711	−0.0532	5.84 × 10^−3^	5.60 × 10^−1^	ns
Pb	BMI	0.0034	−0.0035	0.0103	3.11 × 10^−1^	1	ns
Pb	Age	−0.0012	−0.0033	0.001	2.71 × 10^−1^	1	ns
Pb	SexM	0.0591	−0.0134	0.1317	1.04 × 10^−1^	1	ns
Cr	BMI	0.0008	−0.0069	0.0086	8.28 × 10^−1^	1	ns
Cr	Age	−0.0015	−0.0039	0.0009	1.93 × 10^−1^	1	ns
Cr	SexM	0.0603	−0.0209	0.1416	1.36 × 10^−1^	1	ns
Se	BMI	−0.0019	−0.0054	0.0016	2.70 × 10^−1^	1	ns
Se	Age	−0.0006	−0.0017	0.0005	2.87 × 10^−1^	1	ns
Se	SexM	−0.0304	−0.0674	0.0066	1.01 × 10^−1^	1	ns

**Table 11:** Demographic characteristic of the studied population enrolled in the study.

Gender	*n*	BMI (Range) [kg m^−2^]	%	Min–Max Age	Median Age	Mean Age ± SD
Female	13	22.41(15.97–36.29)	28.89	20.00–80.00	41.00	47.18 ± 19.52
Male	32	25.55(17.36–44.69)	71.11	26.00–86.00	60.00	59.04 ± 14.69
population	45	24.62(15.97–44.69)	100.00	20.00–86.00	56.50	55.21 ± 17.06

Abbrevations: Body mass index (BMI); the standard deviation (SD).

## Data Availability

The original contributions presented in this study are included in the article/Supplementary Materials. Further inquiries can be directed to the corresponding authors.

## Supplementary Materials

The following supporting information can be downloaded at: https://www.mdpi.com/article/10.3390/ijms27062585/s1.
