# Supplementary material for: Distribution of Toxic and Essential Elements in Autopsy Organs of Subjects Living in South-Eastern Poland
Source: Int J Mol Sci. 2026 Mar 11;27(6):2585. doi: 10.3390/ijms27062585 (PMC13026681; doi:10.3390/ijms27062585)
Supplement: Supplementary file 1 [file ijms-27-02585-s001.zip › ijms-4157691-supplementary-conversion.pdf]

**Table S1.** Characteristics of the study population stratified by tissue type.

| Organ | brain | liver | Lungs (average of left and right lung samples) | bronchi |
|---|---|---|---|---|
| Studied population | 39 | 37 | 29 | 28 |
| Females | 13 | 12 | 7 | 8 |
| Males | 26 | 25 | 21 | 20 |
| The mean age | 53.3 years (SD = 17.1) | 53.0 years (SD = 17.3) | 55.2 years (SD = 16.0) | 55.2 years (SD = 16.0) |
| The median age | 55.5 years | 55.0 years | 58.0 years | 58.0 years |
| The age range | 20 to 86 years | 20 to 86 years | 23 to 80 years | 23 to 80 years |
| The age skewness | −0.24 | −0.18 | −0.54 | −0.54 |
| The age kurtosis | −0.67 | −0.72 | −0.53 | −0.53 |
| The mean body mass index (BMI) | 24.8 kg/m² (SD = 5.8) | 24.9 kg/m² (SD = 5.9) | 24.2 kg/m² (SD = 5.2) | 24.2 kg/m² (SD = 5.2) |
| The median of BMI | 24.2 kg/m² | 24.2 kg/m² | 23.9 kg/m² | 23.9 kg/m² |
| The range of BMI | 16.0 to 44.7 kg/m² | 16.0 to 44.7 kg/m² | 16.0 to 36.3 kg/m² | 16.0 to 36.3 kg/m² |
| The BMI skewness | 1.21 | 1.17 | 0.74 | 0.74 |
| The BMI kurtosis | 2.66 | 2.46 | 0.54 | 0.54 |

**Table S2**. Detailed characteristic of the ICP MS method.

| Sample analysis | |
|---|---|
| **ICP MS spectrometer** | PlasmaQuant MS Q (Analytik Jena, Germany) |
| radio frequency (RF) power [kW] | 1.20 |
| argon gas flows [L $min^{-1}$] | 9.0 (plasma), 1.5 (auxiliary), 1.02 (nebulizer) |
| sampling depth [mm] | 5.0 |
| spray chamber type (temp.) | double pass Scott-type (+3°C) |
| scans/replicates | 20/5 |
| dwell time [ms] | 20 |
| polyatomic interferences correction | integrated Collision Reaction Cell (iCRC) |
| collision cell (helium flow) | Al, Cd, Ce, Co, Cu, Dy, Er, Eu, Gd, La, Mn, Nd, Ni, Pr, Sm, Tm, V, Zn (80 mL $min^{-1}$), Ca, Fe, K, Mg, Na, P (150 mL $min^{-1}$) |
| reaction cell (hydrogen flow) | As, Cr, Se (80 mL $min^{-1}$), |
| no gas correction | Be, Ba, Cs, Rb, Sb, Sr, Pb, Tl |
| **Quality control** | |
| internal standard | 5 μg $L^{-1}$ ($^{6}$Li, Sc, Y, Rh, Ir, Bi) |
| control of a series of analyses | blank, reagent blanks, control sample, CRMs |
| uncertainty (k=2) | <10%; including a sample preparation <20% |
| traceability | CRMs, standard addition (recovery 80-120%) |
| certified standard materials | Table S2 |
| method detection limits (min–max) [μg $g^{-1}$] * | Al (0.46–1.4); As (0.009–0.028); Ba (0.054–0.160); Be (0.003–0.009); Ca (2.8–8.4); Cd (0.003–0.009); Ce (0.001–0.004); Co (0.002–0.005); Cr (0.005–0.014); Cs (0.003–0.008); Cu (0.036–0.107); Dy (0.001–0.002); Er (0.001–0.003); Eu (0.001–0.002); Fe (0.70–2.1); Gd (0.002–0.004); K (2.9–8.8); La (0.004–0.011); Mg (1.6–4.7); Mn (0.020–0.061); Na (1.7–5.1); Nd (0.002–0.005); Ni (0.001–0.004); P (3.6–11); Pb (0.005–0.015); Pr (0.001–0.002); Rb (0.009–0.026); Sb (0.013–0.040); Se (0.019–0.056); Sm (0.002–0.004); Sr (0.007–0.021); Tb (0.0003–0.001); Tl (0.001–0.002); Tm (0.004–0.011); V (0.003–0.009); Zn (0.040–0.12); |

* – min-max range of method limit detection, corresponding to max and min sample weight

**Table S3.** The full list of CRMs used to validate the ICP MS measurements.

| Matrix | CRM |
|---|---|
| water | NIST SRM 1643f, CRM-449, CRM-582 |
| soil | NIST SRM 2709, NIST SRM 2709a, AN-ZP01, AN-ZP02 |
| sediment | LGC-6187, IAEA-405, BCR-667, LKSD-1-4, CNS-392 |
| plant | NIST SRM 1515, AN-BM01, NIST SRM 1547, NIST SRM 1573a, NIST SRM 1575a, INCT-OBTL-5, INCT-PVTL-6, BCR-402, |
| wood | NIST SRM 2791, NIST SRM 2790, IPE-240 |
| mushroom | CS-M-3, IPE 120, |
| tissue | BCR-668, DB001, BCR-185R, ERM-BB184, Seronorm L1, DA-120a, BCR-627, |
| food, feed | NIST SRM 1568b, BCR-129, ERM-CD281, AN-BM02, LGC-7162, NIST SRM 1570a, ERM-BD150, INCT-TL-1, INCT-CF-3, INCT-SBF-4 |
| materials | NIST SRM 610, NIST SRM 612, NIST SRM 981 |

**Table S4.** ICP-MS analysis of elemental distribution across the studied tissue samples in μg/g w.w. (wet weight).

| Mn | Ni | Cu | Zn | Cd | Pb | Cr | As | Se |
|---|---|---|---|---|---|---|---|---|
| | | | | **IDL [ng/g]** | | | | |
| 0.035 | 0.0021 | 0.061 | 0.069 | 0.0051 | 0.0087 | 0.0078 | 0.016 | 0.032 |
| | | | | **brain** | | | | |
| 0.1144 | 0.0895 | 7.5447 | 12.2166 | 0.1735 | 0.1764 | 0.1566 | 0 | 0.1588 |
| 0 | 0.5412 | 0 | 5.0786 | 0 | 0 | 2.3905 | 0 | 0 |
| 0.2426 | 0.0125 | 7.9086 | 13.5252 | 0.0178 | 0.1259 | 0.0699 | 0 | 0.2494 |
| 0.3492 | 0 | 0 | 8.9384 | 0 | 0 | 0 | 0 | 0.3037 |
| 2.2049 | 0 | 0 | 35.3988 | 0 | 0 | 0 | 0 | 1.0688 |
| 0.4156 | 0 | 0 | 10.5347 | 0.0741 | 0 | 0 | 0 | 0.2249 |
| 1.3914 | 0 | 0 | 17.1977 | 0 | 0 | 0 | 0 | 0 |
| 0 | 0.5412 | 0 | 5.0786 | 0 | 0 | 2.3905 | 0 | 0 |
| 0.1144 | 0.0895 | 7.5447 | 12.2166 | 0.1735 | 0.1764 | 0.1566 | 0 | 0.1588 |
| 0.2284 | 0 | 3.7385 | 9.1776 | 0.0115 | 0.0566 | 0.0843 | 0 | 0.1667 |
| 0.4289 | 0.0404 | 2.4162 | 7.6174 | 0.018 | 0 | 0 | 0 | 0.1903 |
| 0.4961 | 0.0422 | 9.1198 | 4.1552 | 0 | 0.3806 | 0 | 0 | 0.2253 |
| 0.6196 | 0.0396 | 3.1505 | 4.8461 | 0.0271 | 0 | 0.0354 | 0 | 0.1905 |
| 0.4937 | 0.0181 | 3.2439 | 5.816 | 0.0118 | 0 | 0.1879 | 0 | 0.1807 |
| 0.2949 | 0 | 0 | 8.1329 | 0.029 | 0 | 0.0655 | 0 | 0.1216 |
| 0.4859 | 0.0262 | 22.045 | 11.2256 | 0 | 0.6267 | 0.0701 | 0 | 0.2224 |
| 0.4766 | 0 | 0.6597 | 6.7283 | 0.0071 | 0 | 0.0289 | 0 | 0.1745 |
| 0.5378 | 0 | 5.0338 | 7.6299 | 0.0206 | 0 | 0.0165 | 0 | 0.1833 |
| 0.3026 | 0 | 6.6861 | 9.0302 | 0.0116 | 0.0307 | 0.0177 | 0 | 0.1482 |
| 0.3842 | 0 | 1.9011 | 8.1458 | 0.0092 | 0 | 0.0297 | 0 | 0.1325 |
| 0.3131 | 0 | 14.1908 | 9.7134 | 0 | 0.2223 | 0 | 0 | 0.1508 |
| 0.394 | 0.0565 | 4.2848 | 15.7213 | 0.0046 | 0.0749 | 0.0314 | 0 | 0.1498 |
| 0.1324 | 0 | 4.6893 | 1.6302 | 0.0085 | 0 | 0 | 0 | 0.2344 |
| 0.5516 | 0 | 2.065 | 5.5854 | 0.0101 | 0 | 0 | 0 | 0.2523 |
| 0.1491 | 0 | 0.9821 | 8.8382 | 0 | 0 | 0.0146 | 0 | 0.1665 |
| 0.4474 | 0 | 12.472 | 12.4052 | 0.0057 | 0.3547 | 0.0329 | 0 | 0.2102 |
| 0 | 0 | 10.0341 | 10.7472 | 0 | 0.1535 | 0.022 | 0 | 0.1538 |
| 0.4604 | 0 | 6.4364 | 8.773 | 0.0128 | 1.0925 | 0.0442 | 0 | 0.1473 |
| 0.3968 | 0 | 11.389 | 13.4363 | 0.0184 | 0.167 | 0.0799 | 0 | 0.2006 |
| 0.7413 | 0 | 0 | 9.3377 | 0.0129 | 1.2815 | 0.1116 | 0.0226 | 0.1373 |
| 0.7989 | 0 | 0 | 10.3398 | 0.0493 | 0.2682 | 0.1644 | 0 | 0.2025 |
| 0.1873 | 0 | 0 | 9.3709 | 0.0124 | 0 | 0 | 0 | 0.1638 |
| 0.33 | 0 | 0 | 8.2013 | 0.0481 | 0 | 0.0123 | 0 | 0.1334 |
| 0.1962 | 0 | 9.3371 | 13.503 | 0.0229 | 0 | 0 | 0 | 0.2297 |
| 0.2872 | 0 | 2.4278 | 11.4093 | 0.0285 | 0 | 0.0649 | 0 | 0.2196 |
| 0.1688 | 0 | 0 | 9.298 | 0.0296 | 0 | 0.0591 | 0 | 0.2131 |
| 0.5224 | 0.0224 | 0 | 8.1424 | 0 | 0 | 0.0214 | 0 | 0.1259 |
| 0.4886 | 0 | 0 | 4.1379 | 0.0181 | 0 | 0.0248 | 0 | 0.1877 |
| 0.4828 | 0.0253 | 3.8662 | 10.1235 | 0.0149 | 0 | 0.0364 | 0 | 0.1901 |
| 0.7278 | 0.0069 | 4.8413 | 13.7132 | 0.0291 | 0.0368 | 0.014 | 0 | 0.2411 |
| 0.6092 | 0 | 1.1803 | 9.6757 | 0.0077 | 0 | 0.0402 | 0 | 0.2113 |
| 0.7324 | 0.015 | 2.6826 | 8.4911 | 0.0058 | 0 | 0.0895 | 0 | 0.1748 |
| 0.3512 | 0 | 2.466 | 7.3399 | 0 | 0 | 0.0372 | 0 | 0.378 |
| 0.6998 | 0.0415 | 2.9958 | 11.7387 | 0.0143 | 0.1334 | 0 | 0 | 0.205 |

| | | | | | | | | |
|---|---|---|---|---|---|---|---|---|
| | | | | **Liver** | | | | |
| 3.119 | 0.0014 | 0 | 25.5627 | 1.9511 | 0.0349 | 0 | 0 | 0.4172 |
| 5.3465 | 0.0478 | 0 | 75.8611 | 10.75 | 0.1443 | 0 | 0 | 0.8881 |
| 2.2244 | 0 | 1.5443 | 17.6337 | 0.2419 | 0.1192 | 0 | 0 | 0.2196 |
| 2.1494 | 0.008 | 0.9793 | 20.918 | 1.3722 | 0.1362 | 0.0258 | 0 | 0.2079 |
| 4.3841 | 0.0074 | 2.3218 | 38.2888 | 2.9257 | 0.1154 | 0.0144 | 0.0082 | 0.3105 |
| 5.0832 | 0 | 1.4108 | 32.8169 | 0.2618 | 0 | 1.0098 | 0 | 0.4773 |
| 3.3886 | 0.0167 | 8.5619 | 16.4201 | 0.1364 | 0.0095 | 0 | 0 | 0.6166 |
| 2.2299 | 0 | 4.625 | 25.836 | 1.4937 | 0 | 0.0312 | 0 | 0.3287 |
| 4.1157 | 0 | 1.9817 | 45.4581 | 2.0084 | 0 | 0.0081 | 0 | 0.5519 |
| 3.6502 | 0.1766 | 192.9385 | 147.4304 | 0.9623 | 4.6389 | 0.5218 | 0 | 1.8637 |
| 3.0525 | 0.0325 | 7.9133 | 53.7573 | 0.5827 | 0.3196 | 0.0338 | 0 | 0.5545 |
| 2.7218 | 0.0435 | 6.1513 | 38.5457 | 0.4673 | 0.2032 | 0.0144 | 0 | 0.4426 |
| 1.3249 | 0.0273 | 7.9734 | 15.4466 | 0.2095 | 0.2777 | 0.0568 | 0 | 0.4884 |
| 1.3146 | 0.0011 | 8.3956 | 13.1965 | 0.0821 | 0.0826 | 0.0171 | 0.008 | 0.2477 |
| 3.6337 | 0.0267 | 0 | 34.507 | 0.207 | 0.25 | 0 | 0 | 0.643 |
| 3.0779 | 0 | 6.7985 | 94.2368 | 1.5265 | 0.0202 | 0.0376 | 0 | 0.6482 |
| 4.8341 | 0.1105 | 15.873 | 178.2931 | 0.8542 | 0.2863 | 0.0745 | 0 | 0.6798 |
| 2.4224 | 0 | 4.4703 | 24.7307 | 0.6643 | 0.7855 | 0.017 | 0 | 0.4908 |
| 3.6492 | 0 | 4.7383 | 48.4236 | 1.0131 | 0.7685 | 0.0075 | 0 | 0.504 |
| 6.5598 | 0 | 9.1163 | 75.8474 | 1.355 | 0.2244 | 0.0739 | 0 | 2.0931 |
| 3.4735 | 0 | 1.0162 | 21.0617 | 0.1324 | 0.085 | 0.0068 | 0 | 0.3286 |
| 2.1582 | 0.1347 | 6.9815 | 54.731 | 0.3529 | 0.1981 | 0.018 | 0 | 0.5699 |
| 5.1005 | 0 | 0 | 29.8397 | 0.8344 | 0.1142 | 0.0729 | 0 | 0.3672 |
| 3.9061 | 0 | 6.8168 | 35.5477 | 0.1388 | 0 | 0.0382 | 0 | 0.3867 |
| 3.612 | 0.0359 | 4.7895 | 17.9741 | 0.0544 | 0.2203 | 0.025 | 0 | 0.1905 |
| 4.2366 | 0 | 0 | 51.1494 | 0.2258 | 0.0508 | 0.0252 | 0 | 0.4127 |
| 2.7755 | 0 | 4.236 | 73.9058 | 0.1349 | 0.0544 | 0.0179 | 0 | 0.3952 |
| 2.9135 | 0 | 0 | 77.3686 | 0.1009 | 0.2804 | 0.0309 | 0 | 0.2941 |
| 4.0809 | 0.0156 | 2.3999 | 26.1771 | 0.5161 | 0.0893 | 0.0191 | 0.0091 | 0.3459 |
| 3.7031 | 0.0433 | 2.33 | 30.24 | 0.273 | 0.0685 | 0 | 0 | 0.3934 |
| 1.8772 | 0.0085 | 2.9205 | 32.5213 | 0.1492 | 0.0555 | 0 | 0 | 0.4362 |
| 4.5997 | 0 | 3.5002 | 35.5513 | 1.3265 | 0.0134 | 0.0399 | 0 | 0.5273 |
| 3.0624 | 0.0021 | 2.174 | 42.5382 | 0.4902 | 0.0461 | 0.03 | 0 | 0.4296 |
| 2.767 | 0.0098 | 2.0517 | 28.8961 | 0.0981 | 0.0917 | 0.0141 | 0 | 0.3053 |
| 3.762 | 0.008 | 4.9786 | 32.8477 | 0.1024 | 0.1103 | 0.0184 | 0.0109 | 0.5572 |
| 1.118 | 0.0281 | 4.0876 | 24.895 | 0.0686 | 0.0908 | 0.0177 | 0.0115 | 0.376 |
| 3.2579 | 0.0677 | 1.9855 | 31.6015 | 0.1373 | 0.0469 | 0.0075 | 0 | 0.4643 |
| 2.7029 | 0.365 | 1.979 | 40.0977 | 0.1681 | 0.0835 | 0.0089 | 0 | 0.5253 |
| 2.5853 | 0.5249 | 0.8598 | 29.0359 | 0.0552 | 0.0506 | 0.0123 | 0.0063 | 0.2318 |
| 2.9159 | 0 | 2.6155 | 40.3454 | 0.4255 | 0.0358 | 0.0152 | 0 | 0.6369 |
| 3.7842 | 0 | 4.8925 | 89.462 | 0.7381 | 0.1628 | 0 | 0 | 0.7892 |

| | | | | | | | | |
|---|---|---|---|---|---|---|---|---|
| | | | | **Lungs** | | | | |
| 0.2669 | 0.4607 | 0 | 4.9786 | 0.0703 | 0.4468 | 0.0337 | 0 | 0.1169 |
| 0.381 | 0.1896 | 0 | 7.5293 | 0.0347 | 0.1275 | 0.2734 | 0 | 0.2425 |
| 0.2309 | 0 | 2.0033 | 11.2627 | 0.0383 | 0 | 0.0659 | 0 | 0.1457 |
| 0.2171 | 0 | 0 | 7.1689 | 0.1216 | 0.0711 | 0.0385 | 0 | 0.1447 |
| 0.3356 | 0 | 4.0041 | 8.0133 | 0.115 | 0.0977 | 0.1123 | 0 | 0.1581 |
| 0.1008 | 0 | 0.2673 | 9.391 | 0.0333 | 0 | 0.0383 | 0 | 0.1536 |
| 0.3144 | 0.0492 | 3.1432 | 12.1817 | 0.0972 | 0.1473 | 0.218 | 0 | 0.1899 |

| | | | | | | | | |
|---|---|---|---|---|---|---|---|---|
| 0.1379 | 0.0074 | 2.4577 | 8.2749 | 0.07 | 0.522 | 0.0224 | 0.0093 | 0.2144 |
| 0.1823 | 0 | 0 | 5.7452 | 0.1356 | 0.0781 | 0.0818 | 0 | 0.0989 |
| 0 | 0 | 0 | 5.8483 | 0.3402 | 0 | 0.0208 | 0 | 0.1659 |
| 0.0893 | 0 | 0 | 6.1472 | 0.2947 | 0 | 0.0452 | 0 | 0.1504 |
| 0 | 0 | 0 | 5.508 | 0.2634 | 0.0182 | 0.0243 | 0 | 0.145 |
| 0 | 0 | 0 | 3.7135 | 0.0402 | 0 | 0.0591 | 0 | 0.1376 |
| 0.2163 | 0 | 1.0005 | 10.4279 | 0 | 0.0267 | 0.0056 | 0 | 0.3983 |
| 0.157 | 0.0043 | 0 | 5.6125 | 0.0191 | 0 | 0.0561 | 0 | 0.1407 |
| 0.2291 | 0 | 0 | 8.2115 | 0.3526 | 0 | 0.0333 | 0 | 0.2089 |
| 0.2864 | 0 | 0.8086 | 5.67 | 0 | 0 | 0.0089 | 0 | 0.0842 |
| 0.4249 | 0.0207 | 1.0654 | 16.3532 | 0.0717 | 0.0266 | 0.0094 | 0 | 0.3134 |
| 0.2669 | 0 | 0.2623 | 5.4195 | 0.0056 | 0.024 | 0.1979 | 0 | 0.1847 |
| 0.1581 | 0.0011 | 0.3869 | 7.4126 | 0.0053 | 0.0075 | 0.0538 | 0 | 0.237 |
| 0.3741 | 0.0292 | 0.2382 | 5.3903 | 0.0099 | 0.0672 | 0.139 | 0 | 0.0849 |
| 0.7414 | 0 | 2.2022 | 13.3734 | 0.1236 | 0.0465 | 0.0428 | 0 | 0.2203 |
| 0.1968 | 0.0015 | 0.6845 | 9.0969 | 0.015 | 0.0159 | 0.0353 | 0 | 0.2595 |
| 0.1888 | 0 | 0.7429 | 8.9805 | 0.0705 | 0.0112 | 0.0109 | 0 | 0.1875 |
| 0.1824 | 0 | 0.5491 | 9.5987 | 0.0148 | 0.0129 | 0.0185 | 0 | 0.5166 |
| 0.2085 | 0 | 0.3187 | 9.0782 | 0.0239 | 0.0116 | 0.0657 | 0 | 0.1733 |
| 0.2242 | 0.0409 | 0.4709 | 7.8044 | 0.1428 | 0.0198 | 0.0703 | 0 | 0.1278 |
| 0.3262 | 0.0201 | 0.4393 | 10.8319 | 0.0455 | 0.041 | 0.0435 | 0 | 0.1789 |
| 0.2112 | 0 | 0.0878 | 8.1575 | 0.1572 | 0 | 0.0097 | 0 | 0.23 |
| 0.1486 | 0 | 0.1833 | 7.9709 | 0.2238 | 0 | 0 | 0 | 0.1823 |
| 0.2122 | 0.1618 | 0.2014 | 6.6843 | 0.1685 | 0.0055 | 0 | 0 | 0.2048 |
| 0.1819 | 0.017 | 0.133 | 5.9798 | 0.055 | 0.0105 | 0.0576 | 0 | 0.1014 |
| 0.1962 | 0 | 0.2238 | 6.4895 | 0 | 0 | 0.1143 | 0 | 0.1629 |
| 0.3993 | 0.0221 | 0.2965 | 12.0008 | 0.0268 | 0 | 0 | 0 | 0.24 |
| 0.2066 | 0 | 0.0723 | 8.2795 | 0.1428 | 0.014 | 0.0316 | 0 | 0.1285 |
| 0.1666 | 0.0175 | 0.1038 | 6.4092 | 0.2025 | 0.0069 | 0.0377 | 0 | 0.1578 |
| 0 | 0.0048 | 0.2894 | 7.5667 | 0.0746 | 0.0364 | 0.0608 | 0 | 0.1463 |
| 0.1105 | 0.0069 | 1.3773 | 9.3714 | 0.0252 | 0.0275 | 0.051 | 0 | 0.1457 |
| 0.3211 | 0.0117 | 0.5793 | 8.8996 | 0.6907 | 0.0103 | 0.046 | 0 | 0.1595 |
| 0.2194 | 0.1333 | 0.3468 | 12.5601 | 0.4501 | 0.0285 | 0.0603 | 0 | 0.1845 |
| 0.0851 | 0.0055 | 0.4375 | 8.4318 | 0.4277 | 0.0184 | 0.0575 | 0 | 0.1419 |
| 0.0688 | 0.0315 | 0.3396 | 8.2574 | 0.0229 | 0.0104 | 0.0587 | 0 | 0.1499 |
| 0.3316 | 0.0147 | 0.4744 | 10.5691 | 0.0242 | 0.0203 | 0.0955 | 0 | 0.2064 |
| 0.1323 | 0.0102 | 0.1938 | 5.5357 | 0.1912 | 0.0152 | 0.058 | 0 | 0.1391 |
| 0.1154 | 0.0133 | 0.3365 | 8.9645 | 0.4073 | 0.0216 | 0.0385 | 0 | 0.1805 |
| 0.1123 | 0.0205 | 0.3783 | 5.4711 | 0.1065 | 0.0118 | 0.0273 | 0 | 0.1034 |
| 0.443 | 0.0679 | 4.2812 | 6.9222 | 0.0134 | 0.0335 | 0.0701 | 0.0095 | 0.1635 |
| 0.3048 | 0.0177 | 0.4853 | 7.6114 | 0.1504 | 0.0099 | 0.0533 | 0 | 0.1555 |
| 0.087 | 0.0064 | 0.4586 | 7.861 | 0.1154 | 0.0131 | 0.0223 | 0 | 0.1681 |
| 0.4757 | 0.0127 | 0.4153 | 11.5858 | 0.0262 | 0.0313 | 0.3444 | 0 | 0.2538 |
| 1.609 | 0 | 0.2241 | 6.512 | 0.0092 | 0.0061 | 0.0846 | 0 | 0.1372 |
| 0.2627 | 0 | 0 | 12.9737 | 0.0176 | 0.0599 | 0.1326 | 0 | 0.1591 |
| 0.3931 | 0.0405 | 2.2235 | 5.6436 | 0.036 | 0.0853 | 0.3173 | 0 | 0.098 |
| 0 | 0 | 0.2647 | 8.4838 | 0.0127 | 0 | 0.0796 | 0 | 0.1818 |
| 0.4211 | 0.0132 | 0.3997 | 10.8405 | 0.0259 | 0 | 0.2383 | 0 | 0.2986 |
| 0.2674 | 0.0294 | 3.2409 | 8.5594 | 0.097 | 0.1125 | 0.6297 | 0 | 0.136 |

| | | | | | | | | |
|---|---|---|---|---|---|---|---|---|
| | | | | **bronchi** | | | | |
| 0.1966 | 0 | 5.4821 | 8.6217 | 0.0373 | 0.4585 | 0 | 0.0136 | 0.1269 |
| 0 | 0.0165 | 2.3322 | 9.1209 | 0.1123 | 0.048 | 0 | 0 | 0.1185 |
| 0.1052 | 0 | 2.0209 | 6.1996 | 0.0375 | 0.4571 | 0 | 0.0123 | 0.1518 |
| 0.8899 | 0 | 0 | 10.3535 | 0.2513 | 0 | 0.04 | 0 | 0.1281 |
| 0 | 0 | 0 | 7.3922 | 0 | 0 | 0.0295 | 0 | 0.1083 |
| 0.5795 | 0.0168 | 0.6136 | 8.4095 | 0.0854 | 0.1796 | 0.0385 | 0 | 0.2454 |
| 0.9565 | 0.0211 | 2.2551 | 5.3391 | 0 | 0.0535 | 0.0325 | 0 | 0.1431 |
| 0.3804 | 0.0158 | 1.0924 | 10.1486 | 0.0638 | 0.2984 | 0 | 0 | 0.079 |
| 0.2812 | 0.0451 | 1.2381 | 14.7706 | 0.0675 | 0.0837 | 0 | 0 | 0.1546 |
| 0.2895 | 0 | 0.3589 | 4.9161 | 0.0453 | 0.0082 | 0.0326 | 0 | 0.1465 |
| 0.222 | 0.006 | 0.7998 | 4.7676 | 0.0431 | 0.0383 | 0.0206 | 0.007 | 0.1152 |
| 0.5135 | 0 | 0.51 | 8.0884 | 0.0289 | 0 | 0.0218 | 0 | 0.215 |
| 0.2865 | 0.0186 | 0.939 | 7.4534 | 0 | 0.0623 | 0.0122 | 0.0159 | 0.15 |
| 0.786 | 0.0204 | 5.7606 | 9.2078 | 0.0576 | 0.0778 | 0.0316 | 0 | 0.1227 |
| 0.262 | 0.5856 | 0 | 6.945 | 0.0119 | 0 | 0.0221 | 0 | 0.163 |
| 0.4211 | 0.2636 | 0.1891 | 6.0244 | 0.0103 | 0.008 | 0.0135 | 0 | 0.1632 |
| 0.1285 | 0 | 0.2628 | 4.1378 | 0.0277 | 0.0181 | 0.0091 | 0 | 0.0961 |
| 0.3281 | 0 | 0.1159 | 6.9546 | 0.0255 | 0.0203 | 0 | 0 | 0.2224 |
| 0.2593 | 0 | 0.1031 | 8.776 | 0.0449 | 0.1527 | 0 | 0.0158 | 0.0956 |
| 0.1241 | 0.0017 | 0.1912 | 10.0687 | 0.0377 | 0.1337 | 0 | 0.0183 | 0.186 |
| 0.1364 | 0.0023 | 0 | 13.8104 | 0.0426 | 0.2853 | 0 | 0 | 0.1344 |
| 0.122 | 0.0013 | 0 | 6.1602 | 0.0446 | 0.079 | 0 | 0 | 0.0792 |
| 0.2362 | 0.0059 | 0.1191 | 4.2328 | 0.02 | 0 | 0.331 | 0 | 0.23 |
| 0.495 | 0 | 0.2399 | 10.6594 | 0.1855 | 0.0432 | 0 | 0 | 0.2518 |
| 0.2024 | 0 | 0.4752 | 5.3054 | 0.1276 | 0.0112 | 0 | 0.0047 | 0.1429 |
| 0.2234 | 0.007 | 0.1892 | 4.9644 | 0.0438 | 0.027 | 0.0137 | 0 | 0.1427 |
| 0.1853 | 0.0199 | 0.1963 | 4.7861 | 0.0374 | 0.0208 | 0.0241 | 0 | 0.1092 |
| 0.1893 | 0.011 | 0.1352 | 3.4497 | 0.0327 | 0.0066 | 0.0115 | 0.0061 | 0.1024 |

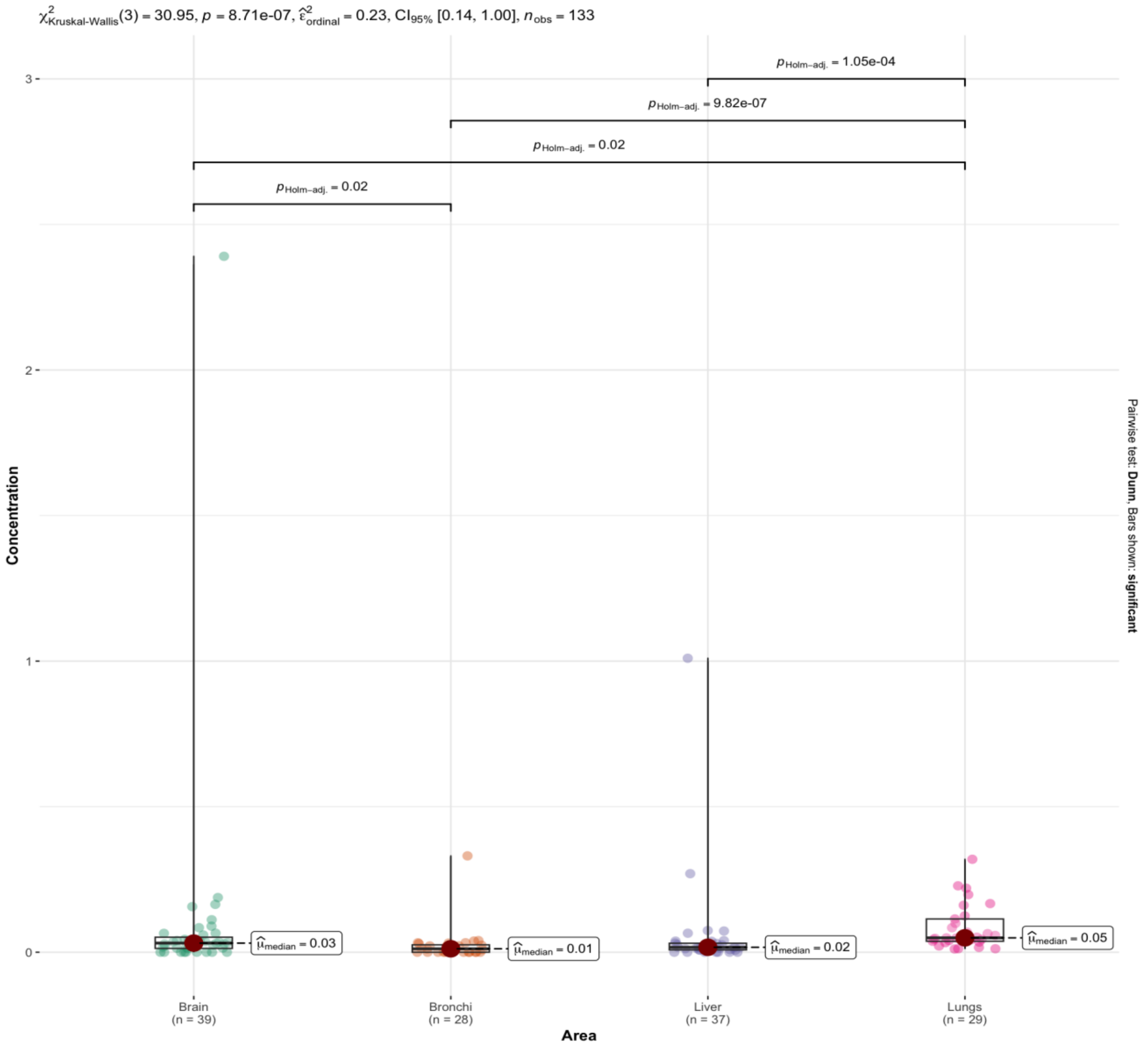


**Figure S1.** Comparative analysis of Cr concentrations across tissues. Results are presented as box-and-whisker plots (median, IQR, and range) with overlaid individual data points. Horizontal brackets indicate pairwise Dunn's post-hoc comparisons, with corresponding Holm-adjusted p-values displayed above. The global Kruskal-Wallis test result is provided as an overall summary of variance.

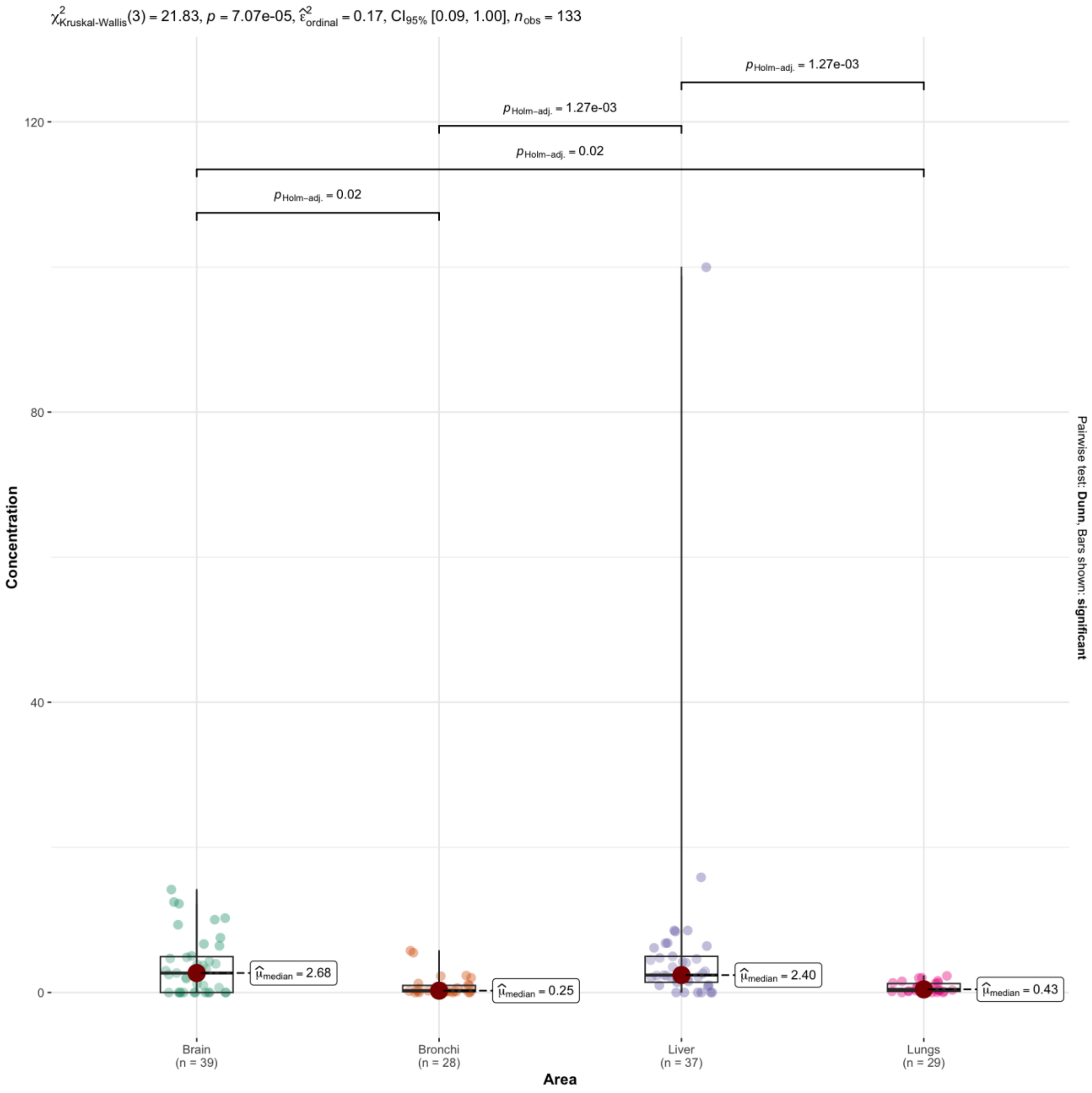


**Figure S2**. Comparative analysis of Cu concentrations across tissues. Results are presented as box-and-whisker plots (median, IQR, and range) with overlaid individual data points. Horizontal brackets indicate pairwise Dunn's post-hoc comparisons, with corresponding Holm-adjusted p-values displayed above. The global Kruskal-Wallis test result is provided as an overall summary of variance.

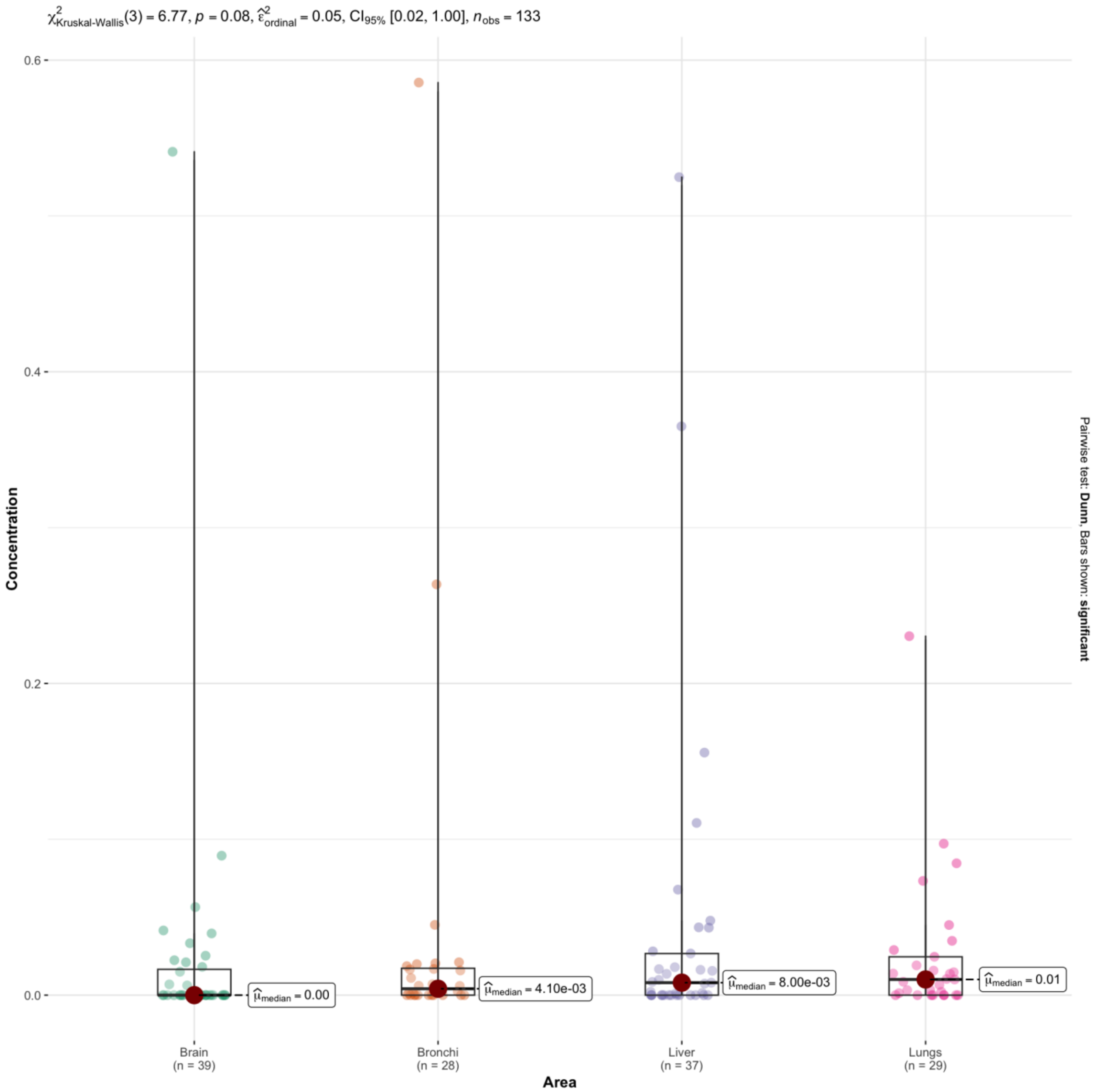


**Figure S3.** Comparative analysis of Ni concentrations across tissues. Results are presented as box-and-whisker plots (median, IQR, and range) with overlaid individual data points. Horizontal brackets indicate pairwise Dunn's post-hoc comparisons, with corresponding Holm-adjusted p-values displayed above. The global Kruskal-Wallis test result is provided as an overall summary of variance.

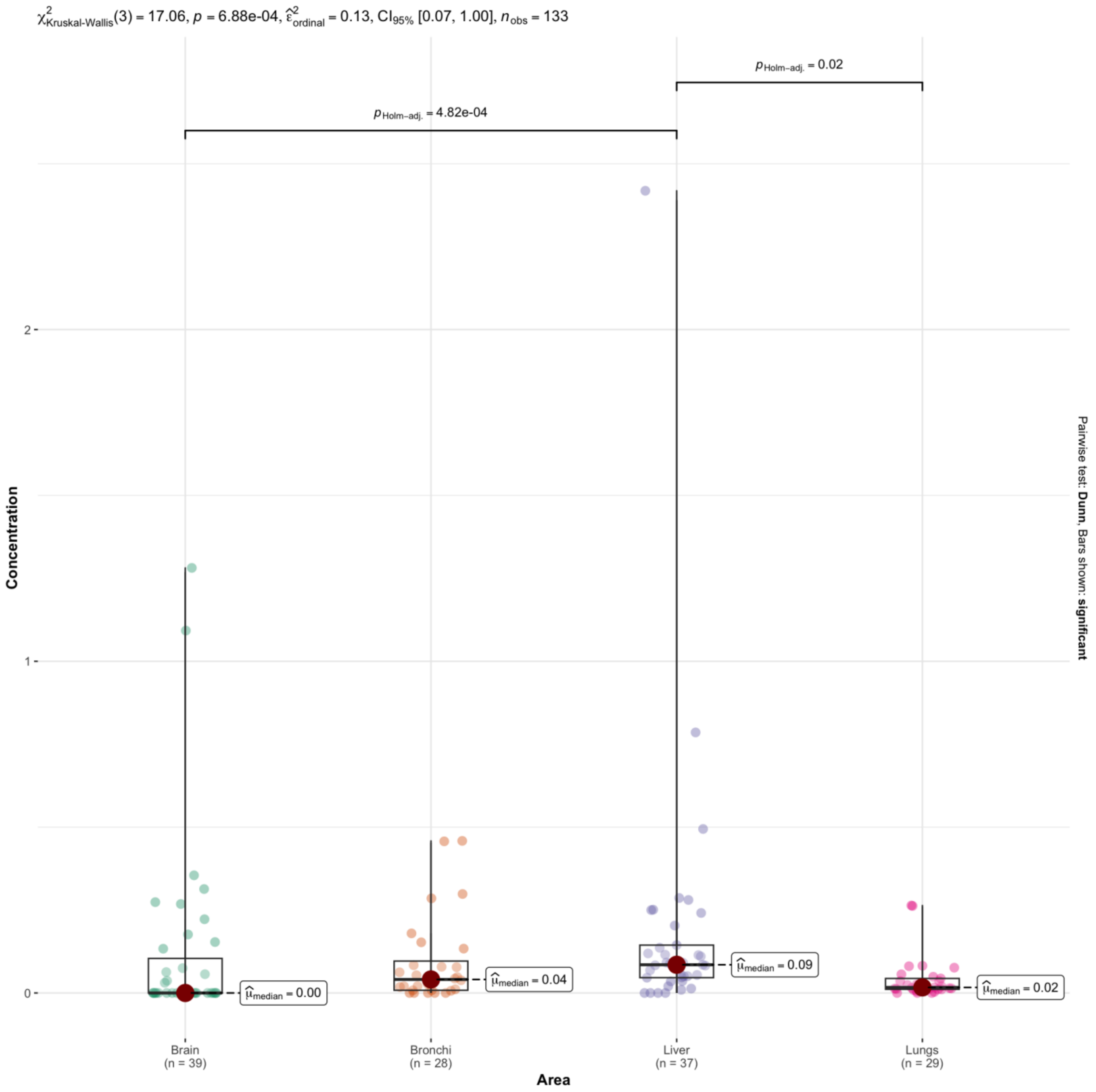


**Figure S4.** Comparative analysis of Pb concentrations across tissues. Results are presented as box-and-whisker plots (median, IQR, and range) with overlaid individual data points. Horizontal brackets indicate pairwise Dunn's post-hoc comparisons, with corresponding Holm-adjusted p-values displayed above. The global Kruskal-Wallis test result is provided as an overall summary of variance.
